# Conformational stability of hemocyanins regulates their lysosomal and proteasomal degradation, influencing their pro-inflammatory effects on mammalian antigen-presenting cells

**DOI:** 10.3389/fimmu.2025.1603070

**Published:** 2025-12-01

**Authors:** Michelle L. Salazar, Claudia d’Alençon, Diego Díaz-Dinamarca, Javier Bustamante, Byron Castillo, Alejandra Alvarado, Fabián Salazar, Augusto Manubens, María Inés Becker

**Affiliations:** 1 Fundación Ciencia y Tecnología para el Desarrollo (FUCITED), Santiago, Chile; 2 Facultad de Ciencias Químicas y Farmacéuticas, Universidad de Chile, Santiago, Chile; 3 Pathology Advanced Translational Research Unit (PATRU), Department of Pathology and Laboratory Medicine, Emory University School of Medicine, Atlanta, GA, United States; 4 Medical Research Council Centre for Medical Mycology, University of Exeter, Exeter, United Kingdom; 5 Investigación y Desarrollo, Biosonda S.A., Santiago, Chile

**Keywords:** hemocyanins, oligomeric glycoproteins, antigen-presenting cells, intracellular degradation, immunomodulation, lysosome, immunoproteasome, protein based-adjuvant

## Abstract

**Introduction:**

Mollusk hemocyanins are known for their immunomodulatory properties in mammals. Their applications include serving as carrier glycoproteins, functioning as protein-based adjuvants, and acting as non-specific immunostimulants in cancer vaccine strategies. Their immunomodulatory effects are attributed to their xenogenicity, structural complexity, high molecular mass, and glycosylations. Recent studies have begun to clarify the immunological mechanisms by which hemocyanins induce: multiligand properties arising from interactions with C-type lectins and Toll-like receptors, and the promotion of a Th1 immune response. However, the subsequent effects of hemocyanins, particularly their intracellular targeting and degradation kinetics, remain poorly understood. The present study is the first to comprehensively examine the processing of two well-characterized hemocyanins, known for their conformational stability and clinical significance: KLH from *Megathura crenulata* and CCH from *Concholepas concholepas*.

**Methods:**

We correlated their degradation with the kinetics of the proinflammatory response they induce and their subcellular localization using the JAWS II cell line and bone marrow-derived dendritic cells (BMDCs). We utilize OVA to highlight the differences between this protein and the hemocyanins.

**Results:**

The results showed that KLH and CCH induced significant TNF levels after 24 hours and also promoted the secretion of IL-6 and IL-12p40 after 96 hours, along with the upregulation of CD80 and CD86. This delayed response corresponds with their slow intracellular degradation. Colocalization studies using LAMP-1 demonstrated that hemocyanins were localized to lysosomes only after prolonged stimulation, suggesting that they are likely stored in intracellular depots. Furthermore, hemocyanins were shown to colocalize with LMP-2 and a4, indicating that they undergo processing in the proteasome. In contrast, OVA displayed faster degradation with mild pro-inflammatory effects within 24 hours. Pharmacological inhibition of lysosomal cathepsins or the proteasome reduced the hemocyanin-dependent secretion of IL-6 and IL-12p40. Additionally, fragmented hemocyanins led to lower, less sustained cytokine levels compared to their native form.

**Conclusions:**

These findings emphasize that hemocyanins, owing to their complex oligomeric structure and high stability, are slowly processed by APCs, thereby contributing to their immunogenicity. This property is particularly relevant when hemocyanins are used as carriers for vaccine antigens because their delayed kinetics can enhance the magnitude, quality, and persistence of responses.

## 1 Introduction

Hemocyanins are complex copper-binding glycoproteins primarily involved in oxygen transport in the hemolymph of some mollusks (1). These glycoproteins have gained significant attention in biomedicine and biotechnology because of their immunomodulatory properties when administered to mammals (2, 3). Hemocyanins promote a T helper type 1 (Th1) immune response, which is essential for cell-mediated immunity against intracellular pathogens and cancer cells. Consequently, hemocyanins have been utilized as protein-based adjuvants (PBAs) in vaccines, as carriers of haptens, and as nonspecific immunostimulants in cancer immunotherapy (4, 5). The first hemocyanin characterized, keyhole limpet hemocyanin (KLH) from the *keyhole limpet* (*Megathura crenulata*), has been widely used as a carrier for antibody production and as an antitumor molecule in superficial bladder cancer. Moreover, KLH is being evaluated in 14 early-phase trials as an investigational medicinal product (IMP), mainly targeting T-cell activation (6). However, the clinical use of KLH is limited by non-standardized doses, unoptimized immune response endpoints, and an incomplete understanding of its specific immunological effects (7).

The widespread applications of KLH have prompted the exploration of other alternative gastropod hemocyanins, such as CCH from *Concholepas concholepas* (8), FLH from *Fissurella latimarginata* (9), RtH from *Rapana thomasiana* (10), HtH from *Haliotis tuberculata* (11), HlH from *Helix lucorum* (12), and PcH from *Pomacea canaliculata* (13). These alternative hemocyanins exhibit comparable and even superior immunological and antitumor properties, as demonstrated in murine and human cell cultures, as well as in animal models of oral cancer, melanoma, prostate cancer, colon carcinoma, myeloid tumor, and superficial bladder cancer (9, 14–22). Notably, CCH and KLH have been evaluated as a safe protein adjuvant in phase I/II clinical trials for therapeutic vaccines involving autologous antigen presenting cells (APCs) loaded with allogeneic tumor cell lysates (23–26). Despite the promising applications of hemocyanins as PBAs and immunostimulants, the precise immune mechanisms behind their effects are still not fully understood.

The beneficial immune effects of hemocyanins are partially attributed to their structural features. Hemocyanins are high-molecular mass glycoproteins (4–8 MDa) self-assembled into cylinders of 35 nm in diameter; this complex structure, known as didecamer, is comprised of twenty subunits (350–450 kDa), each of which includes, in turn, eight globular domains named functional units (FUs) (27–29). Moreover, hemocyanins are highly glycosylated, reaching up to 4.5% (w/w), with mannose, fucose, and N-acetylglucosamine being the predominant oligosaccharides.; These glycosylations play a crucial role in their immune-modulatory functions in mammals (9, 30–35). For instance, KLH induces the maturation of dendritic cells (DCs) via a mannose receptor (MR)-dependent mechanism (36). Moreover, chemically and enzymatically deglycosylated KLH triggers a decreased proinflammatory response in murine macrophages and bone marrow-derived DCs (BMDCs), as well as in human monocyte-derived DCs (moDCs) (9, 33, 37). Similarly, N-deglycosylation of mollusk hemocyanins decreased the humoral response in a melanoma murine model (33). These results were significant but partial, suggesting that hemocyanins activate the immune response through multiple pathways. Indeed, CCH and KLH are multiligands of C-type lectin receptors (CLRs) such as MR and Dendritic Cell-Specific Intercellular Adhesion Molecule-3-Grabbing Nonintegrin (DC-SIGN) and of Toll-like receptors (TLRs) such as Toll-like receptor 4 (TLR4) (5, 37, 38). Furthermore, APCs internalize hemocyanins through receptor-mediated endocytosis and macropinocytosis, with MR and DC-SIGN facilitating their uptake and contributing to IL-6 secretion in moDCs (37). However, the subsequent effects of hemocyanins as complex glycoproteins with structural stability, such as their intracellular targeting and degradation kinetics, remain insufficiently understood (30, 39–41).

The intracellular degradation of exogenous proteins results in the generation of an immunopeptidome, which consists of peptides that bind to the major histocompatibility complex (MHC-I, MHC-II) and non-classical molecules, thereby activating T cells (42). In the lysosomal pathway, cathepsins mediate controlled proteolysis within acidic compartments, while in the proteasomal pathway, polyubiquitinated proteins are unfolded and degraded in the cytosol (43). Additionally, proinflammatory stimuli can upregulate the expression of the LMP2, LMP7, and LMP10 catalytic subunits, leading to the assembly of the immunoproteasome (44). Variations in intracellular conditions, such as localization, redox environment, and pH, can accelerate protein unfolding; however, protein half-life varies from a few minutes to several days, depending on their intrinsic stability (45). It is important to note that mollusk hemocyanins exhibit significant intrinsic stability: KLH shows a higher melting temperature and free energy of stabilization in its oligomeric form compared to the isolated subunits (39). Moreover, oligomerization and oxygen-binding increase the conformational stability of CCH (46). Similarly, high concentrations of chaotropic agents are required to disrupt the structures of FLH and KLH. This treatment disrupts the quaternary structure of these hemocyanins and significantly decreases their immunogenic effects *in vitro* and *in vivo* studies (33). In contrast, KLH, RvH, and HtH exhibit high stability in their reassociation under ionic conditions (47). These findings demonstrate that hemocyanins possess high conformational stability. This characteristic plays a crucial role in determining the intracellular fate of antigens during processing in APCs, from their uptake to the final presentation of antigenic peptides, influencing immunogenicity and immune polarization (45). Thus, T cell priming and antibody responses may be dependent on the proteolytic processing; this has been demonstrated using two unrelated model antigens, RNAse and horseradish peroxidase, which were not immunogenic in their fixed, undigestible forms (48). Moreover, a particulate form of Ovalbumin (pOVA) promoted a stronger IFNγ response compared to native OVA in an OT-II model, and these effects were attributed to the slower degradation of OVA compared to pOVA by APCs (49). Furthermore, lysozyme with high stability elicited minimal immune responses, suggesting that hyperstable proteins are not processed for adequate immune presentation (50). However, these model antigens are smaller than 45 kDa, highlighting the need for complementary studies on how APCs process and respond to complex, high-molecular mass, and oligomeric glycoproteins, such as hemocyanins. To date, the use of FLH as an adjuvant for OVA in an OT-I model has shown that T-cell priming is decreased when lysosomal acidification is impaired (5). Although these results suggest that lysosomal degradation plays a role in the adjuvant activity of hemocyanins, further comprehensive studies regarding hemocyanin intracellular processing are needed.

In a previous investigation, we analyzed the processing of CCH and OVA in BMDCs. The immunoblot results showed that after 72 hours of culture, a band of 49 kDa of CCH was observed, indicating that the BMDCs were unable to process CCH completely. In contrast, at this time, OVA was fully degraded. However, this study did not correlate these findings with the kinetics of pro-inflammatory cytokines released during this period or with the subcellular organelles involved in the processing (41). In this context, we aim to investigate further the correlation between the proinflammatory immune response and the kinetics of proteolytic degradation of hemocyanins in two murine APC models, considering the contributions of both lysosomes and proteasomes. We use OVA, as previously mentioned, to highlight the contrast between this protein and hemocyanins (51, 52).

CCH and KLH were selected as model hemocyanins because of their well-documented efficacy and safety as adjuvants in clinical trials. BMDCs were used as standardized professional APCs (38, 41). The JAWS II cell line was also employed due to its potential in molecular applications as an alternative model for BMDCs, particularly in terms of the expression of activation markers and cytokine secretion (53). Results demonstrated a correlation between the proinflammatory effects and the kinetics of intracellular proteolysis of CCH and KLH, showing significant degradation after prolonged incubation (96 hours) in both types of APCs. Moreover, CCH and KLH upregulated the costimulatory molecules CD80 and CD86 on the APC cell surface and colocalized with lysosomes for 96 hours. Notably, colocalization with immunoproteasomes was observed earlier and persisted throughout 96 hours. Pharmacological inhibition of lysosomal and proteasomal pathways reduced the long-term proinflammatory responses, and partial proteolysis of hemocyanins modified cytokine secretion kinetics, supporting that hemocyanin processing influences their immunogenicity. Additionally, partially digested hemocyanins resulted in lower and less sustained cytokine levels compared to the native form, reinforcing the notion that structural integrity is crucial for their immunomodulatory capacity.

Altogether, our findings support the significance of the hemocyanins’ structural complexity in their proinflammatory effects during the early stages of the immune response, providing valuable insights into the immunological response induced by these complex oligomeric glycoproteins.

## 2 Materials and methods

### 2.1 Hemocyanins and ovalbumin

Hemocyanin from *Concholepas concholepas* (CCH) was provided by Biosonda Corp., (Santiago, Chile), and it was purified as described by De Ioannes et al. (8) in sterile and endotoxin-free conditions. As indicated by Jiménez et al. (38), endotoxin content was assessed using a PyroGene Recombinant Factor C Endotoxin Detection Assay kit (Lonza Group, Walkersville, MD, USA); the endotoxin level was undetectable. Hemocyanin from *Megathura crenulata* (KLH) was obtained from Merck Millipore (*Massachusetts*, MA, USA) as an endotoxin-free solution (<0.5 EU/mg). Ovalbumin (OVA) was purchased from InvivoGen (San Diego, CA, USA) in sterile and endotoxin-free conditions.

Fluorescent-labeled hemocyanins and OVA were obtained as described by Villar et al. (37). Briefly, proteins were diluted in dissociation buffer (0.2 mM PBS, 0.1 M sodium bicarbonate), and Alexa594 (Thermo Scientific, Waltham, MA, USA) was added according to the datasheet instructions. The reaction was stopped after 1 hour by adding hydroxylamine, and the labeled proteins were washed with PBS pH 7.2 using Amicon Ultra-4 systems (Merck Millipore). The final concentration of the conjugated proteins was determined using Pierce Bradford 660 according to the manufacturer instructions and considering bovine serum albumin (both from Thermo Scientific) as the protein standard.

Partially digested hemocyanins were prepared as described by Oliva et al. (54). Briefly, CCH and KLH were digested with porcine pancreatic elastase (Sigma-Aldrich) in PBS pH 7.2 at 37°C for 2 hours. Digestion was stopped by heat inactivation at 95°C for 5 minutes. To confirm the proteolysis, the resulting hemocyanins, CCHel and KLHel, were evaluated by SDS-PAGE as described by De Ioannes et al. (8).

All protein solutions were prepared using water for human irrigation (Baxter Healthcare, Charlotte, NC, USA) and analytical-grade material. Before cell stimulation, solutions were filtered through 0.2µm membrane filters (Millipore, Billerica, MA, USA).

### 2.2 Mice

C57BL7/6 wild-type (WT) mice were obtained from Universidad de Chile (Santiago, Chile) and bred at Biosonda. The study was conducted by the Guidelines for the Care and Use of Laboratory Animals of the Agencia Nacional de Investigación y Desarrollo (ANID), Chile (FONDECYT Project 1201600), and the FUCITED Bioethics Committee. Animal experiments were performed using male and female mice following the animal care and welfare protocols approved by FUCITED (Ethical Approval No. 06/2015), in compliance with all relevant local ethical regulations.

### 2.3 Cell culture of JAWS II cells and bone marrow-derived dendritic cells

JAWS II, a murine cell line established from bone marrow cells of a p53-knockout C57BL/6 mouse, was cultured in Minimum Essential Medium alpha (MEMα), supplemented with 10% fetal bovine serum (FBS), 4 mM L-glutamine, 1 mM penicillin/streptomycin and 1 mM sodium pyruvate (All from HyClone, Logan, UT, USA). GM-CSF (5 ng/mL, Miltenyi Biotec, Bergisch Gladbach, Germany) was added to promote cell differentiation into an immature dendritic cell (iDC) phenotype.

Bone marrow-derived dendritic cells (BMDCs) were obtained as described by Lutz et al. (52) and modified by Jiménez et al. (38). Briefly, 2×10^6^ bone marrow cells from the tibia and femur from C57BL/6 mice were seeded in culture dishes in Roswell Park Memorial Institute 1640 medium (RPMI-1640) supplemented with 10% FBS, 2 Mm L-glutamine, 1 mM penicillin/streptomycin (all from HyClone) and 20 ng/mL GM-CSF (Miltenyi Biotec). Cells were differentiated for 8 days and then collected by pipetting. BMDCs and JAWS II were carefully maintained at 37 °C and 5% CO_2_.

### 2.4 Surface receptors and costimulatory molecule analyses by flow cytometry

To analyze the basal expression of receptors relevant to hemocyanin-induced immunity, unstimulated JAWS II or BMDCs were prepared for flow cytometry. Cells (5x10^5^ cells/condition) were stained using the following antibodies: anti-CD11c-PE, anti-MR-FITC, anti-TLR4-PE, anti-SIGNR1-Alexa647, and anti-SIGNR5-Alexa488 (all from BioLegend, San Diego, CA, USA). EFluor780 (Thermo Scientific) was used to select the live population. To analyze hemocyanin incorporation, APCs were incubated during 1 hour with CCH- and KLH-Alexa594 (10 μg/mL). Samples were washed with PBS-FBS 2% and fixed in PBS-paraformaldehyde (PFA) 2%. Cells were acquired using a BD FACSDiva Flow Cytometer.

Regarding costimulatory molecules, JAWS II or BMDCs were seeded in 24-well culture plates (5x10^5^ cells/well). Cells were stimulated with CCH, KLH, OVA (0.5 mg/mL), LPS (1 ng/mL), or equivalent volumes of PBS from 24 to 96 hours. Cells were collected by pipetting and stained using the following antibodies: anti-CD80-Alexa488, anti-CD86-Alexa647, anti-MHC-I-PE, and anti-MHC-II-Alexa647 (all from BioLegend). EFluor780 (Thermo Scientific) was used to select the live population. Cells were washed with PBS-FBS 2% and fixed in PBS-PFA 2%. Cells were acquired using a BD FACSDiva Flow Cytometer.

### 2.5 Cytokine quantification by ELISA and flow cytometry

IL-12p40 was quantified by ELISA as previously standardized by our group, with modifications (33). Briefly, JAWS II or BMDCs were seeded onto 24-well culture plates (5x10^5^ cells/well) and stimulated with CCH, KLH, or OVA (0.5 mg/mL) for 24, 72, and 96 hours. LPS (Enzo Life Sciences, Long Island, NY, USA, 1 ng/mL) was used as the positive control. Cells treated with PBS, the vehicle of hemocyanins, were used as the negative control. Supernatants were analyzed using a BD OpTEIA kit (BD Biosciences, NJ, USA) according to manufacturer instructions. Results were collected as the optical density (OD) at 450 nm using a SynergyHTX plate reader.

For multiplex quantification, we used a Cytometric Bead Array Kit (CBA) (BD Biosciences), according to Villar et al., with modifications (37). Briefly, JAWS II or BMDCs were seeded onto 96-well culture plates (1x10^5^ cells/well) and stimulated with native hemocyanins (CCH, KLH), partially digested hemocyanins (CCHel, KLHel), or OVA (0.5 mg/mL) for 24, 72, and 96 hours. The same positive and negative controls, LPS and PBS, were used. Culture supernatants were collected and stored at -80°C until analysis. Samples were prepared for flow cytometry according to the manufacturer’s instructions and acquired using a BD FACSDiva Flow Cytometer.

### 2.6 Cell viability assay by colorimetric staining

JAWS II or BMDCs were seeded in 96-well culture plates (5x10^4^ cells/well). Cells were stimulated with CCH, KLH, OVA (0.5 mg/mL), LPS (1 ng/mL), or an equivalent volume of PBS for 24, 48, 72, 96, and 120 hours. Cells were stained with AlamarBlue™ (Thermo Scientific) as instructed by the manufacturer. The 570 and 600 nm OD values were obtained using a Synergy HTX plate reader.

### 2.7 Hemocyanin processing kinetics by Western Blotting analyses

JAWS II or BMDCs were seeded in 6-well culture plates (5x10^5^ cells/well) and stimulated with CCH, KLH, and OVA (0.5 mg/mL) from 24 to 96 hours.

Cell lysis and immunoblotting were performed as described by Jimenez et al. (38). Briefly, cell cultures were lysed using RIPA solution and a protease inhibitor (both from Thermo Scientific). Protein concentration was determined using Bradford 660 (Thermo Scientific) according to the manufacturer’s instructions. For immunoblotting, 40 μg of total protein was added to gradient polyacrylamide gels (5-15%). Electrophoresis was performed for 4 hours at 80 Volts, and the transfer was conducted overnight at 10 Volts at 4°C. The nitrocellulose membranes were blocked in Tris-buffered saline (TBS) with 1% BSA for 2 hours. Then, membranes were incubated with polyclonal anti-CCH and anti-KLH antibodies produced by our laboratory to detect proteolytic fragments (54). For the OVA, an anti-OVA serum (Abcam, Cambridge, UK) was used. A monoclonal anti-β-actin antibody (Abcam) was used as a control for the technique. Primary antibodies were incubated overnight, then the membranes were stained with specific anti-IgG sera coupled to alkaline phosphatase (Thermo Scientific). Membranes were washed 3 times after every step with TBS-Tween 0,2%. The experiments were revealed by adding the substrate NBT-BCIP (Thermo Scientific).

### 2.8 Hemocyanin intracellular localization by immunofluorescence

JAWS II or BMDCs were seeded in 24-well culture plates (5x10^5^ cells/well). Cells were stimulated with CCH, KLH, or OVA coupled to Alexa594 (10 μg/mL), from 24 to 96 hours. After incubation, cells were collected by gentle pipetting, centrifuged for 5 minutes at 1,600 rpm, and seeded onto Nunc 8-well immunofluorescence chambers at a density of 4x10^4^ cells/well. Of note, chambers were previously treated with poly-L-lysine (Cytiva, HyClone) for 2 hours at 37 °C to promote cell adhesion to coverslips.

Samples were stained as described by Villar et al. (37). Briefly, cells were fixed with PBS-PFA 4% for 20 minutes and permeabilized with PBS-Tritón X-100 0,5% for 10 minutes. Then, samples were blocked with PBS-BSA 5% for 30 minutes and stained with primary antibodies to detect LAMP1 (Abcam), α4, and LMP2 (both from Santa Cruz, Dallas, TX, USA). Two hours later, cells were incubated with specific anti-rat IgG (H+L)-FITC serum and with DAPI (both from Thermo Scientific). All steps were carried out at room temperature. Samples were washed with PBS three times at each step. Nunc chambers were mounted using 7 μL/well of a DABCO solution (1,4-Diazabicyclo[2.2.2]octane solution) provided by Biosonda Corp. Samples were analyzed by confocal microscopy using a Zeiss LSM700 microscope.

### 2.9 Pharmacological inhibitors of proteasomal and lysosomal pathways

MG132 and epoxomicin were used as reversible and irreversible proteasome inhibitors, respectively. Leupeptin and pepstatin A were used as inhibitors of lysosomal cathepsins. Bafilomycin A1 was used as an inhibitor of lysosomal acidification (56–61). These inhibitors have been characterized to study antigen processing of model proteins, such as OVA, and details are provided in **Table 1**.

**Table 1:** Pharmacological inhibitors of lysosomal and proteasomal processing.

Inhibitor	Pathway	Target	Effect on target	Reference(s)
MG-132	Proteasomal	Proteasome active site	Reversible inhibitor of the ubiquitin-proteasome pathway, blocking the activity of the 26S proteasome.	(51, 52)
Epoxomicin	Proteasomal	Catalytic proteasome subunits	Irreversible inhibitor which targets the proteasome's chymotrypsin-like (CT-L) activity.	(53)
Bafilomycin A1	Lysosomal	v-ATPase	Inhibitor of vacuolar-type H(+)-ATPase, inhibits acidification in lysosome.	(54)
Leupeptin	Lysosomal	Cathepsins B, H and K active site	Membrane-permeable protease reversible inhibitor that works by potently inhibiting serine, cysteine, and threonine proteases, including enzymes like cathepsin B, trypsin, and calpain.	(55)
Pepstatin	Lysosomal	Cathepsin D active site	Inhibition of aspartyl-protease activity, including pepsin, cathepsin D and cathepsin E.	(56)

JAWS II or BMDCs were seeded onto 24-well culture plates (5x10^5^ cells/well). Cells were pretreated with each inhibitor (0–50 nM) or an equivalent volume of dimethylsulfoxide (DMSO, Merck), the vehicle of the inhibitors, for 30 minutes before stimulation with hemocyanins. To assess the proinflammatory response, cytokines were quantified from supernatants using ELISA and CBA as previously described, with the IC_50_ values obtained in the standardization experiment for pretreatment of cells 30 minutes before hemocyanin stimulation (0.5 mg/mL). Hemocyanin processing after 96 hours was also analyzed as previously described by immunoblotting.

### 2.10 Data processing and statistical analysis

Flow cytometry files were analyzed using FlowJo 7.6.5; data processing included gating of live and single cells, analyses of mean fluorescence intensity (MFI) values, and percentage of positive cells for the abovementioned markers as reported by Jiménez et al. (38). CBA data was obtained from the Flow Cytometry service from Facultad de Ciencias, Universidad de Chile and reported as the concentration of each cytokine (pg/mL). ELISA data were obtained as the OD, and values were interpolated into standard curves to determine cytokine concentration (pg/mL) (33). Principal Component Analysis (PCA) of cytokine profiles was performed using RStudio 4.2.3 functions, and clustering was assessed by two approaches: K-means and hierarchical clustering. The latter was chosen as the most suitable method based on the data structure. Clustering results were visualized using the *factoextra* function, which displayed the groups formed in the PCA space.

Confocal imaging files were analyzed using Fiji from the Image J software; data processing included obtaining Mander’s coefficients in regions of interest (ROI), including the different z-stacks (37). Western Blot photographs were analyzed using ImageJ; data processing including integrated density and normalization of hemocyanin or OVA values against the signal from control β-actin and background (62).

For all experiments, graphs were created using GraphPad Prism 10.4 and RStudio 4.2.3, showing data as mean ± standard deviation (SD) from three independent experiments unless otherwise stated in the figure legends. Data were analyzed as nonparametric, nonpaired, using the U-Mann Whitney (for comparisons between 2 groups) or the Kruskal-Wallis tests (for comparisons among several groups), considering p<0.05 as significant.

## 3 Results

### 3.1 CCH and KLH, unlike OVA, induce a delayed proinflammatory response by APCs

**Figure 1:**
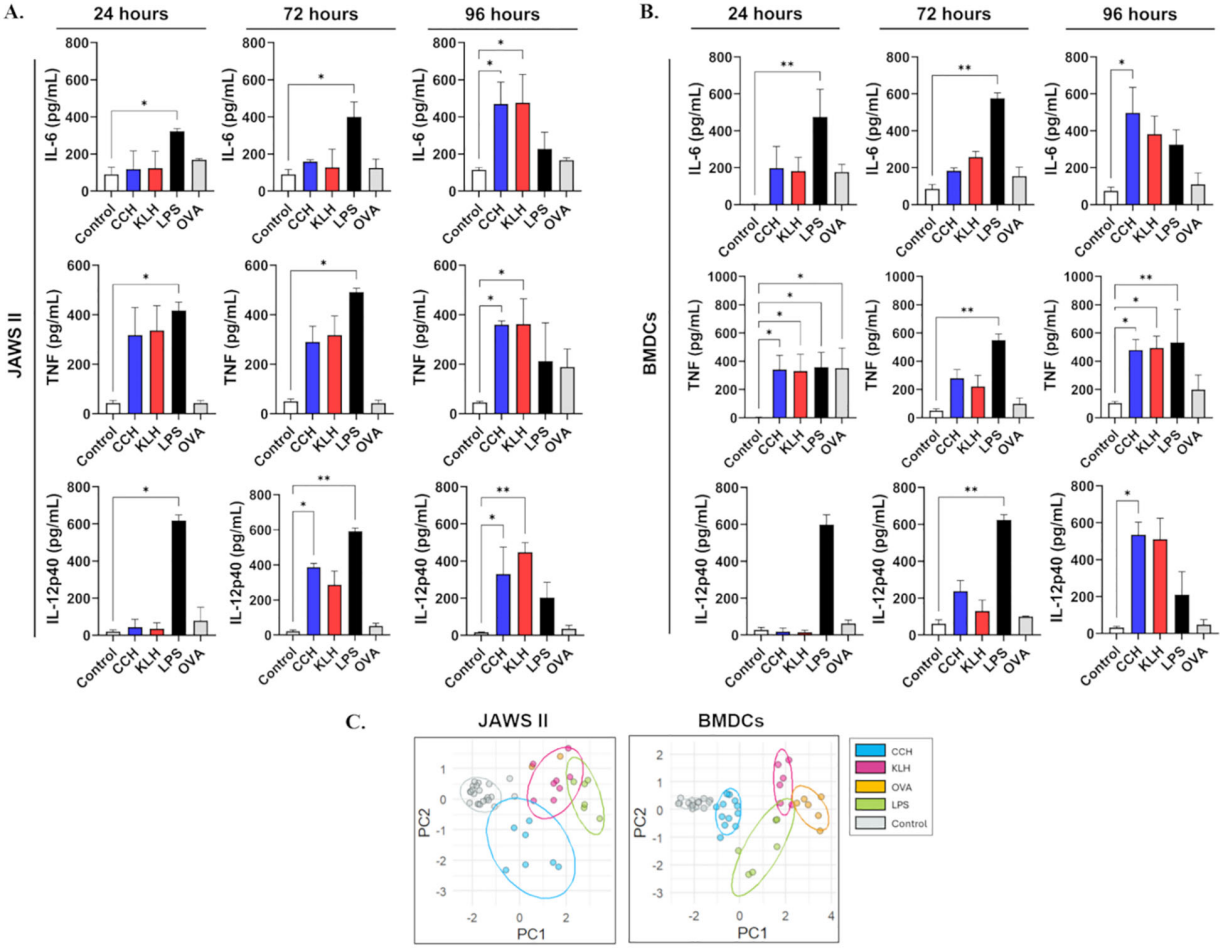
CCH and KLH promote a delayed proinflammatory response in APCs. JAWS II cells **(A)** or BMDCs **(B)** (2x10^5^) were stimulated with CCH (blue) and KLH (red) at 0.5 mg/mL. LPS (black, 1 ng/mL) served as the positive control and OVA (grey, 0.5 mg/mL) was included as a model antigen. Unstimulated cells were used as the negative control (Control, white). Supernatants were collected after 24, 72, and 96 hours of stimulation, and cytokines were quantified using a cytometric bead array (CBA) kit or BD OptEIA kits (ELISA). Data represent the mean ± SD of four independent experiments, analyzed by the Kruskal-Wallis test, where the samples were compared against the negative control. *p<0.05, **p<0.01. **(C)** Principal Component Analysis (PCA) and subsequent hierarchical clustering analysis of cytokine secretion profiles (IL-6, TNF, IL-12p40, IL-2, IL-4, IL-10, IL-17A, and IFNγ). The PCA plot displays each sample as a point in two-dimensional space defined by the first (PC1) and second (PC2) principal components.

First, we evaluated the effects of CCH and KLH on cytokine secretion kinetics over 96 hours and compared them with OVA. We analyzed several cytokines, including IL-2, IL-4, IL-6, IL-10, IL-12p40, IL-17A, IFN-γ, and TNF. This study aims to complement previous findings, which were primarily limited to measuring IL-6 and IL-12p40 for up to 24 hours in mice (9, 38). Interestingly, our results indicated that the proinflammatory effects of hemocyanins peaked at 96 hours in JAWS II cells, with IL-6, TNF, and IL-12p40 levels comparable to those induced by LPS, the positive control (**Figure 1A**). IFN-γ, IL-2, IL-4, IL-10, and IL-17A were also quantified, but they were not detected in any condition or were detected at low levels (<5 pg/mL) (**Supplementary Figure 1A**). The proinflammatory response induced by LPS was sustained over 96 hours, while OVA showed no significant effects. As expected, LPS resulted in the secretion of IL-6, IL-12p40, and TNF, whereas OVA had modest effects on IL-6, which was detectable only up to 24 hours.

In BMDCs, both CCH and KLH triggered comparable cytokine responses compared to JAWS II (**Figure 1B**). At 24 hours, CCH and KLH stimulated the production of TNF and IL-6. OVA induced these cytokines to a lesser extent or failed to induce them throughout the experiment, in contrast to LPS. At 72 hours, CCH also induced IL-12p40 in addition to the previously observed TNF and IL-6. By 96 hours, the levels of IL-6, TNF, and IL-12p40 induced by hemocyanins peaked, comparable to those observed with LPS stimulation. IL-2, IL-4, IL-17A, IL-10, and IFNγ were detected at low levels or below the detection limit (**Supplementary Figure 1B**). Time points beyond 96 hours were excluded because of a significant decrease in cell viability at 120 hours post-stimulation with hemocyanins and OVA; notably, LPS-treated cells exhibited cytotoxicity at 72 hours. (**Supplementary Figures 2A, B**).

To further explore the differences in the proinflammatory effects among CCH, KLH, LPS, and OVA, we performed principal component analysis and clustering based on cytokine secretion data. Analyses revealed that CCH and KLH form distinct clusters, suggesting that they elicit distinct immune signatures, despite their similar proinflammatory kinetics. (**Figure 1C**). OVA and LPS also clustered separately. Furthermore, the PCA allowed us to identify which cytokines contribute the most to the separation among groups (**Supplementary Table 1**). Results were comparable for JAWS II and BMDCs, where TNF, IL-6, and IL-12p40 showed strong positive loadings on PC1, supporting their role as key drivers of the immune profiles. In contrast, IL-2, IL-4, IL-17A, IL-10, and IFNγ exhibited negative loadings on PC2 or moderate loadings on PC1 and/or PC2.

Our results showed that CCH and KLH, unlike OVA, trigger a prolonged proinflammatory milieu with comparable kinetics but differential signatures. Moreover, JAWS II and BMDCs exhibited similar but not identical responses, consistent with the expression of TLR4, MR, and the murine orthologs of DC-SIGN, SIGNR1, and SIGNR5 by BMDCs. In contrast, JAWS II exhibited high SIGNR1 and SIGNR5 expression, with modest levels of TLR4 and no MR on the cell surface (**Supplementary Figures 3A, B**).

### 3.2 CCH and KLH enhance the late upregulation of co-stimulatory molecules and MHC expression on APCs

**Figure 2:**
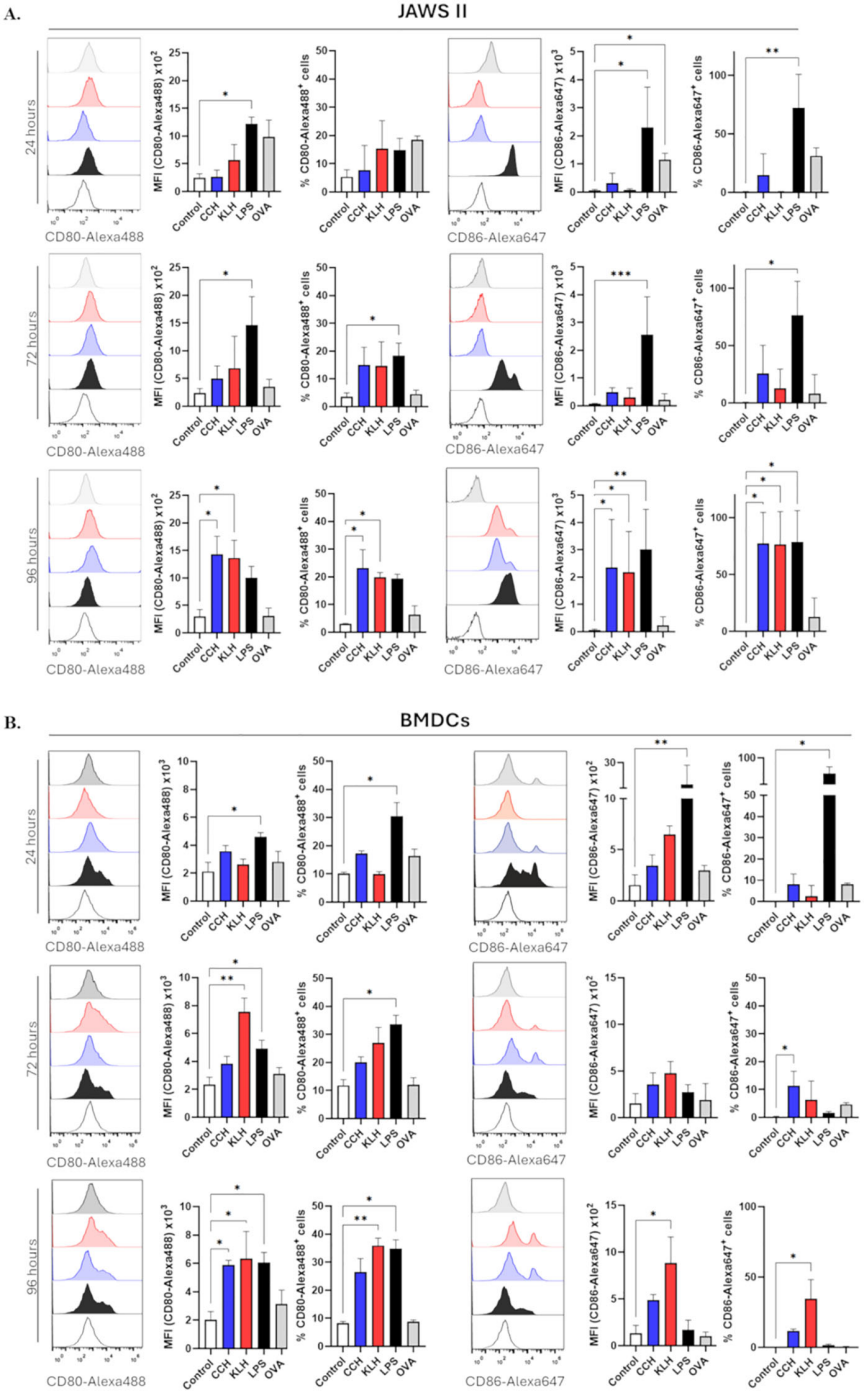
CCH and KLH promote the upregulation of CD80 and CD86 on APCs. JAWS II **(A)** or BMDCs **(B)** (2x10^5^) were stimulated with CCH (blue), KLH (red), or OVA (grey) at a final concentration of 0.5 mg/mL. LPS (black, 1 ng/mL) was used as the positive control. Unstimulated cells were used as the negative control (Control, white). Cells were stained with anti-CD80-Alexa488 or anti-CD86-Alexa647 antibodies after 24, 72, and 96 hours. EFluor780 viability dye was used to select live cells. Data show the mean ± SD of four independent experiments. Statistical analyses were performed using the Kruskal-Wallis, where the samples were compared against the negative control (Control white) and *p<0.05, **p<0.01. ***<0.001.

To further investigate the differential kinetics between hemocyanins and OVA, we analyzed the effects of CCH and KLH on the expression of co-stimulatory molecules (CD80, CD86) and major histocompatibility complex (MHC) molecules (MHC-I and MHC-II) in JAWS II cells and BMDCs after 24 to 96 hours. Unlike the early induction observed with OVA and LPS as positive control, both CCH and KLH resulted in a late upregulation of CD80 and CD86 in both cell types, with peak expression at 96 hours (**Figures 2A, B**). Specifically, we observed increases in the percentage of CD80- and CD86-positive cells, concomitant with higher mean fluorescence intensity (MFI) values compared to the controls. Notably, the increase in CD80 and CD86 was observed only at this late point in JAWS II, while it was already detectable at 72 hours in BMDCs. In contrast, OVA induced only modest effects after 24 hours. This confirms that hemocyanins, rather than other small proteins, promote late upregulation of these co-stimulatory molecules.

Moreover, both CCH and KLH were found to induce MHC-II in JAWS II; however, we did not observe any detectable differences in MHC-I levels, likely due to the already high baseline levels (**Supplementary Figure 4A**), the characteristics of this cell line (53). In contrast, both LPS and OVA-induced MHC-II responses occurred earlier than those of CCH and KLH, with OVA showing a more transient effect. In BMDCs, both CCH and KLH upregulated MHC-I and MHC-II expressions at 96 hours, while OVA did not significantly affect MHC-I levels (**Supplementary Figure 4B**). These findings suggest that both CCH and KLH modulate the expression of key molecules involved in antigen presentation, exhibiting distinct kinetics compared to LPS, a non-protein TLR4 agonist, and OVA, a small protein antigen.

### 3.3 Hemocyanins exhibit a delayed colocalization with lysosomes in APCs, in contrast to OVA

Given the differences observed in the immune profiles of hemocyanins compared to OVA, we focused on understanding how APCs process hemocyanins. Specifically, we investigated their colocalization with lysosomes, which are essential for generating the immunopeptidome in APCs and are among the most well-characterized proteolytic compartments (42, 63). The colocalization of CCH or KLH with the lysosomal marker LAMP1 was analyzed through indirect immunofluorescence (IFI) over a 24 to 96-hour time course in JAWS II and BMDCs.

**Figure 3:**
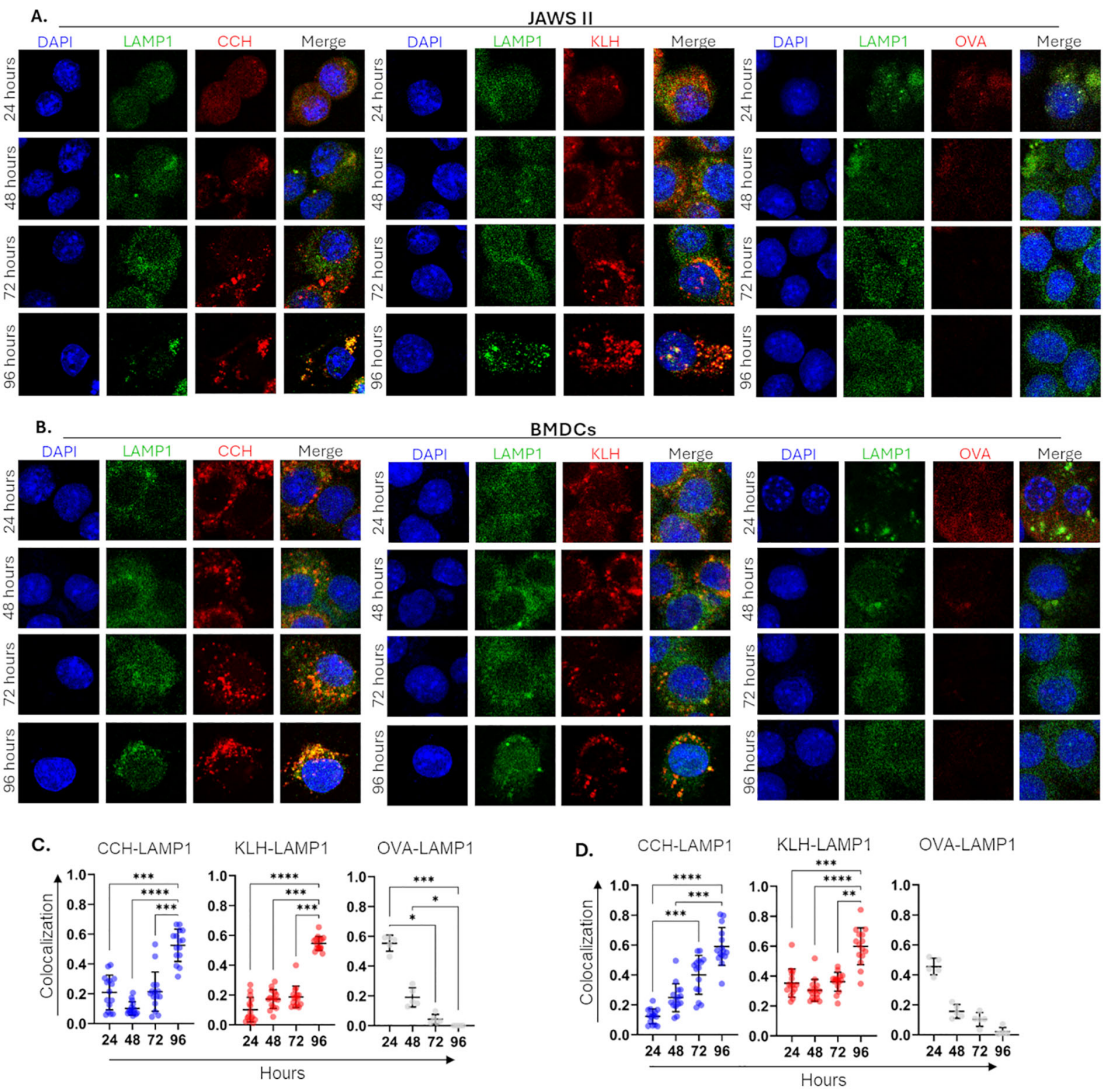
Colocalization of hemocyanins with LAMP1 in APCs indicates late lysosomal processing. JAWS II cells **(A, C)** or BMDCs **(B, D)** (1x10^5^) were stimulated with Alexa594 labelled CCH, KLH, and Ovalbumin (OVA) (red). After 24–96 hours, cells were collected, and 4x10^4^ live cells were seeded in poly-lysine-treated cover slips. Samples were fixed, permeabilized, blocked, and incubated with a rat monoclonal anti-LAMP1 antibody and a secondary anti-rat-IgG-FITC antibody (green). DAPI was used to localize nuclei (blue). Upper panels **(A, B)** show representative microscopy images. Quantification of colocalization **(C, D)** was performed by Mander’s coefficient estimation using Fiji from ImageJ. Data show the mean ± SD from at least 15 Alexa594^+^ FITC^+^ cells in three independent experiments; superimposed points represent individual cells. Statistical analyses were performed using the Kruskal Wallis, where *<0.05, **p<0.01, ***p<0.001, ****p<0.0001.

CCH and KLH conjugated to Alexa594 (red fluorescence) were detectable by IFI at all time points in JAWS II cells, whereas OVA-Alexa594 was detectable until 48 hours (**Figure 3A, C**). While hemocyanins were compartmentalized, LAMP1 was distributed diffusely throughout the cells, except for its clustering observed at 24 hours in OVA-treated cells or at 96 hours in CCH- and KLH-treated cells. Mander’s coefficient indicated a significant increase in the colocalization of CCH and KLH with LAMP1 at 96 hours with a mean coefficient of 0.58. In comparison, OVA showed partial colocalization at 24 hours with a mean coefficient of 0.52, which decreased to <0.2 after 48 hours. Fewer cells could be analyzed in OVA conditions beyond 48 hours, as the protein was detected in only a limited number of cells.

In BMDCs, CCH exhibited a time-dependent increase in colocalization with LAMP1, as indicated by a mean Mander’s coefficient that increased from 0.11 at 24 hours to 0.67 at 96 hours (**Figures 3B, D**). Similarly, KLH exhibited significant colocalization with LAMP1 at 96 hours with a mean coefficient of 0.71. Conversely, OVA showed partial colocalization at 24 hours, with a mean coefficient of 0.57, with decreased values below 0.18 thereafter.

In summary, CCH and KLH are localized to lysosomes only after prolonged stimulation, exhibiting a kinetic profile similar to the proinflammatory response observed in both APC models.

### 3.4 Hemocyanins colocalize with the proteasomal markers *α*4 and LMP2, depending on the type of APC

**Figure 4:**
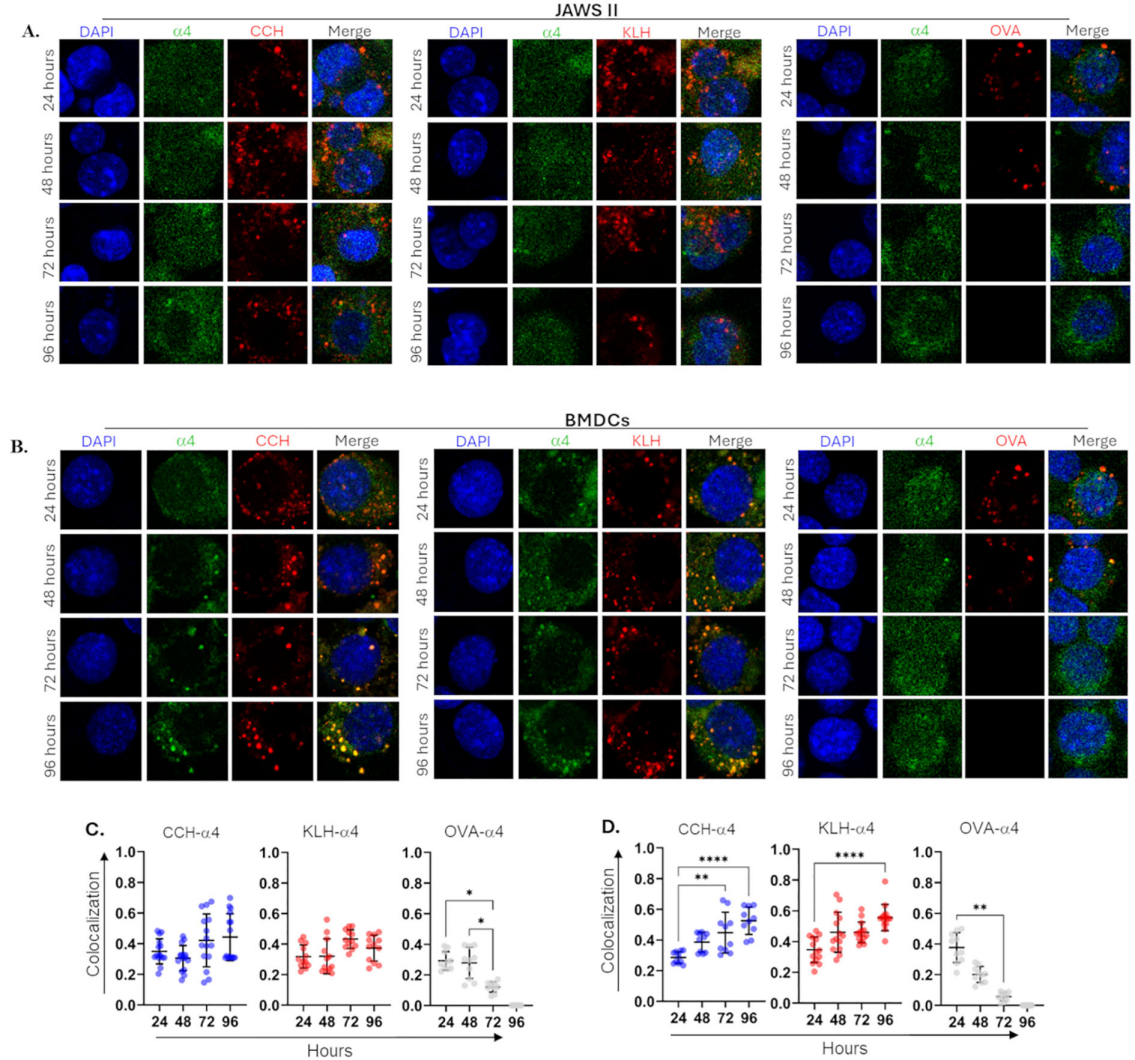
Colocalization of CCH and KLH with α4 suggests their degradation by proteasome in BMDCs but not by JAWS II. JAWS II **(A, C)** or BMDCs **(B, D)** (1x10^5^) were stimulated with CCH, KLH, and OVA coupled to Alexa594 (red). After 24–96 hours, cells were collected, and 4x10^4^ live cells were seeded on poly-lysine-treated cover slips. Samples were fixed, permeabilized, blocked, and incubated with a rat monoclonal anti-α4 antibody. Cells were then treated with an anti-rat-IgG-FITC (green) antibody and DAPI (blue). Upper panels **(A, B)** show representative microscopy images. Quantification of colocalization **(C, D)** was performed by Mander’s coefficient estimation using Fiji from ImageJ. Data show the mean ± SD from at least 15 Alexa594^+^ FITC^+^ cells in three independent experiments; superimposed points represent individual cells. Statistical analyses were performed by Kruskal-Wallis, where the samples were compared against 24 hours post-incubation and *<0.05, **p<0.01, ****p<0.0001.

Lysosomal degradation is essential for proteolysis of exogenous antigens; however, slowly processing proteins may also be targeted for proteasomal degradation (43). Considering the complexity and structural stability of hemocyanins, we examined the colocalization of CCH or KLH with the 20S proteasomal marker α4 under the same conditions as those used to study colocalization with lysosomes (**Figures 4A-D**).

In JAWS II cells, the distribution of the constitutive proteasome was uniform, showing no signs of polarization or redistribution in response to hemocyanins (**Figure 4A**). Additionally, there was no significant increase in colocalization between CCH or KLH and α4 even after 96 hours, with mean coefficients consistently below 0.5. OVA also did not colocalize with α4, with values below 0.3 (**Figure 4C**). In contrast, α4 exhibited a distinct distribution in BMDCs, particularly after prolonged stimulation with CCH and KLH (**Figure 4B**). Notably, significant colocalization between α4 and hemocyanins was observed at 96 hours, with mean values >0.5 (**Figure 4D**). No colocalization was observed with OVA. These findings indicate differences between the two APC models, where BMDCs displayed a kinetic pattern similar to lysosomal colocalization and proinflammatory response.

**Figure 5:**
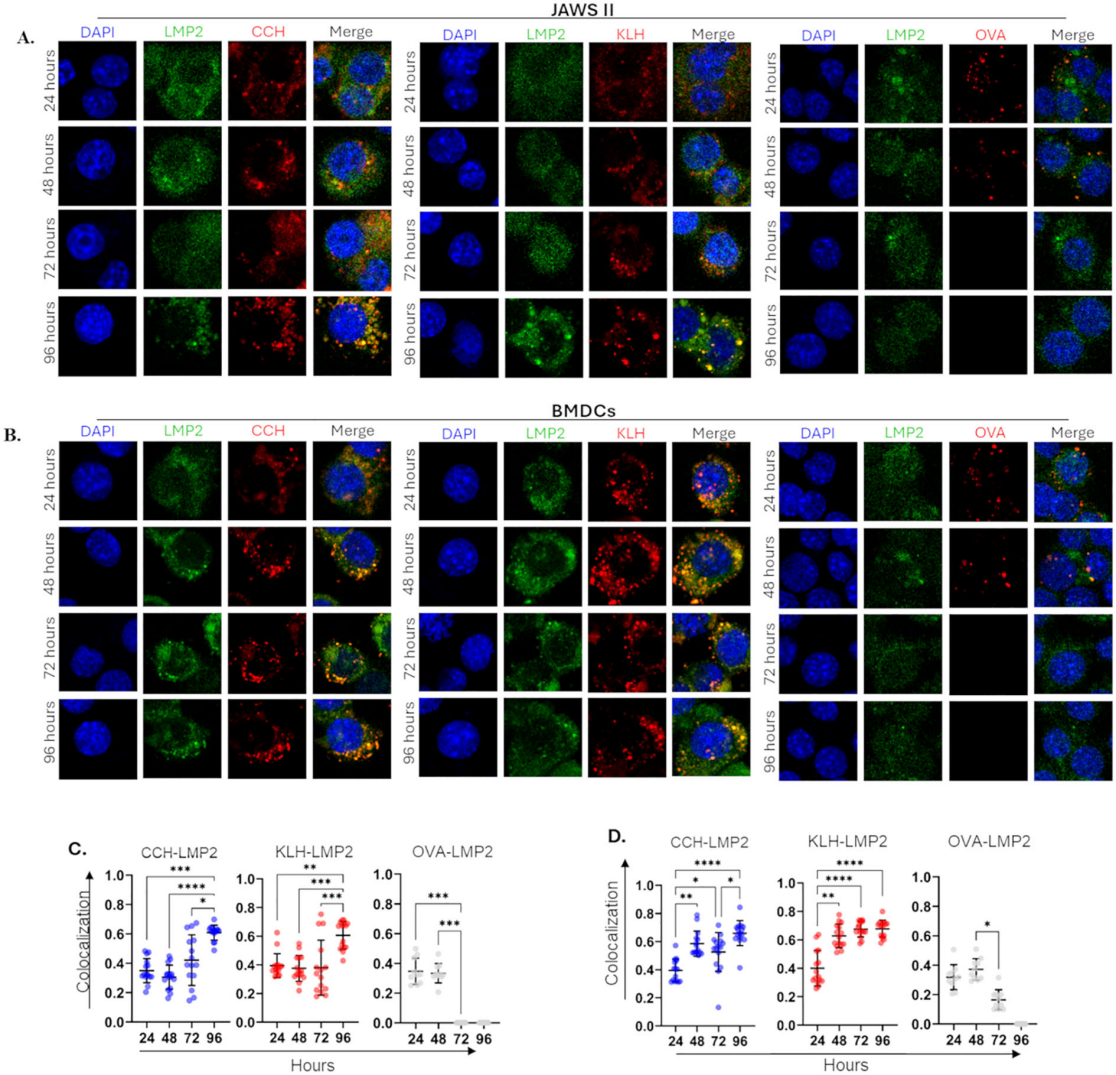
CCH and KLH, unlike OVA, colocalize with the immunoproteasomal catalytic subunit LMP2 in APCs. JAWS II **(A, C)** or BMDCs **(B, D)** (1x10^5^) were stimulated with CCH, KLH, and OVA coupled to Alexa594 (red). After 24–96 hours, cells were collected, and 4x10^4^ live cells were seeded in poly-lysine-treated cover slips. Samples were fixed, permeabilized, blocked, and incubated with a rat monoclonal anti-LMP2 antibody. Cells were then treated with an anti-rat-IgG-FITC (green) antibody and DAPI (blue). Upper panels **(A, B)** show representative microscopy images. Quantification of colocalization **(C, D)** was performed by Mander’s coefficient estimation using Fiji from ImageJ. Data show the mean ± SD from at least 15 Alexa594^+^ FITC^+^ cells in three independent experiments; superimposed points represent individual cells. Statistical analyses were performed by Kruskal-Wallis, where the samples were compared against 24 hours post-incubation, and *<0.05, **p<0.01, ***p<0.001, ****p<0.0001.

**Figure 6:**
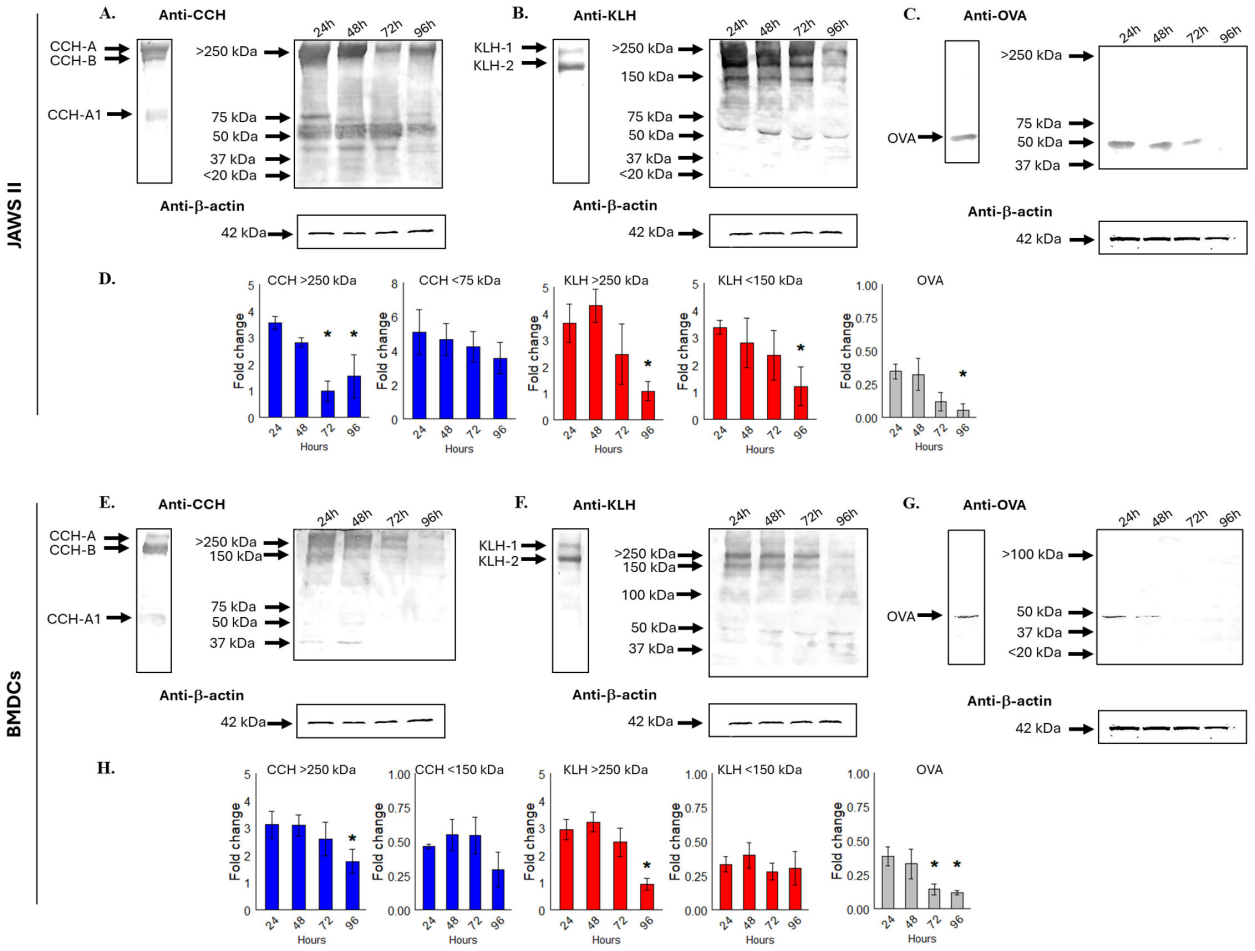
APCs slowly proteolyze CCH and KLH. JAWS II **(A-D)** or BMDCs **(E-H)** (1x10^6^) were stimulated with CCH **(A, D)**, KLH **(B, E)**, and Ovalbumin (OVA, grey) **(C, F)** at 0.5 mg/mL. After 24–96 hours, cell lysates were analyzed by Western Blot using polyclonal mouse anti-CCH, anti-KLH, and anti-OVA antibodies. Membranes were incubated with an anti-rabbit-IgG-AP and revealed using NBT-BCIP. The integrated density was obtained using ImageJ and normalized against the constitutive protein β−actin (fold change). Figures show representative membranes **(A-C, E-G)**, while bar graphs **(D, H)** show the mean ± SD from four independent experiments. Statistical analyses were performed using the Kruskal-Wallis, where the samples were compared against 24 hours post-incubation and *p<0.05.

When exposed to proinflammatory stimulation, APCs upregulate specific proteasomal subunits to assemble the immunoproteasome, which exhibits enhanced chymotrypsin- and trypsin-like activities (44). One upregulated subunit is LMP2, also known as β1i, which was used as a marker in this study (**Figures 5A**–**6D**). IFI analyses on JAWS II revealed a change in LMP2 localization after 96 hours of stimulation with CCH and KLH (**Figure 5A**). Significant colocalization with hemocyanins was observed with mean coefficients above 0.65. No colocalization was noted with OVA (**Figure 5C**).

Strikingly, in BMDCs, we observed a notable reorganization of LMP2 in regions where hemocyanins are concentrated (**Figure 5B**). High colocalization values (above 0.7) were recorded between CCH or KLH and LMP2, with this effect becoming significant as early as 48 hours and persisting up to 96 hours (**Figure 5D**). No colocalization was detected between LMP2 and OVA. Our results showed differences in the proteolytic capacity of the JAWS II cell line and BMDCs after hemocyanin treatment. These findings indicate that hemocyanins are localized to both lysosomes and immunoproteasomes, and the degree of colocalization varies depending on the type of APC used.

### 3.5 APCs exhibit slow processing of CCH and KLH

Given their late proinflammatory effects and delayed colocalization with lysosomes and proteasomes, we next characterize the intracellular processing kinetics of CCH and KLH in JAWS II cells and BMDCs. We assume that the documented conformational stability of hemocyanins is an important factor that can affect their movement within endosomal compartments, a phenomenon also observed with other antigens (48–50, 64). Immunoblotting analysis of JAWS II cells stimulated with CCH or KLH for 24 to 96 hours revealed numerous heterogeneous proteolytic fragments. The main bands were found in fractions of high-molecular mass (>150 kDa), and in fractions smaller than 75 kDa (**Figure 6A**). The results showed that the largest fragments of CCH and KLH were detectable even at 96 hours. However, there was a partial reduction in intensity, as shown by densitometric analysis normalized to the constitutive protein β-actin (fold change in **Figure 6B**). In contrast, the smaller fragments did not show significant changes over time. Notably, OVA appeared as a faint band up to 72 hours, indicating its complete degradation by 96 hours (**Figures 6C, D**).

Similar results were observed in BMDCs, where we identified comparable amounts of proteolytic patterns (**Figures 6E-G**). The larger fragments of CCH and KLH decreased significantly after 96 hours, although this decrease was only partial. The smaller fragments showed no significant differences over time (**Figure 6H**). OVA was detected as a single band for up to 48 hours, indicating that proteolysis occurred more rapidly in BMDCs compared to JAWS II cells (**Figure 6G**).

Overall, these results confirm that APCs process hemocyanins slowly, with larger proteolytic fragments persisting even at 96 hours. This slow processing differs from the rapid degradation of smaller proteins such as OVA.

### 3.6 Pharmacological inhibition of lysosomal cathepsins and the proteasome decreases hemocyanin proinflammatory response in APCs

The intracellular localization of CCH and KLH indicates that both lysosomal and proteasomal pathways are involved in their degradation. To investigate whether inhibiting these pathways affects cytokine secretion, we evaluated the impact of inhibitors targeting lysosomal cathepsins and the proteasome. Five pharmacological inhibitors were selected based on dose-response curve values and cell viability standardization (**Table 1**, **Supplementary Figures 5A, B**). Proteasomal inhibitors MG132 and epoxomicin, as well as lysosomal inhibitors leupeptin and pepstatin, were not cytotoxic at concentrations up to 40 nM. Bafilomycin induced cell death above 20 nM. Moreover, working curves were in the nanomolar (nM) range. Estimated IC_50_ values in JAWS II were 28.7 nM for MG132, 26.1 nM for epoxomicin, 10.5 nM for leupeptin, and 4.8 nM for pepstatin. IC_50_ values were the same for CCH and KLH. Bafilomycin could not be estimated due to nonspecific effects. On the other hand, IC_50_ values in BMDCs were 27.4 nM for MG132, 40 nM for epoxomicin, 38.9 nM for bafilomycin, 11.3 nM for leupeptin, and 21.5 nM for pepstatin using CCH, and 12.1 nM for MG132, 12.7 nM for epoxomicin, 9.9 nM for bafilomycin, 13.5 nM for leupeptin, and 8.5 nM for pepstatin using KLH. Additionally, we assessed the effect of these inhibitors on both basal and LPS-induced cytokine secretion to evaluate any potential nonspecific effects, which were detected only in JAWS II using bafilomycin (**Supplementary Figures 6A, B**).

**Figure 7:**
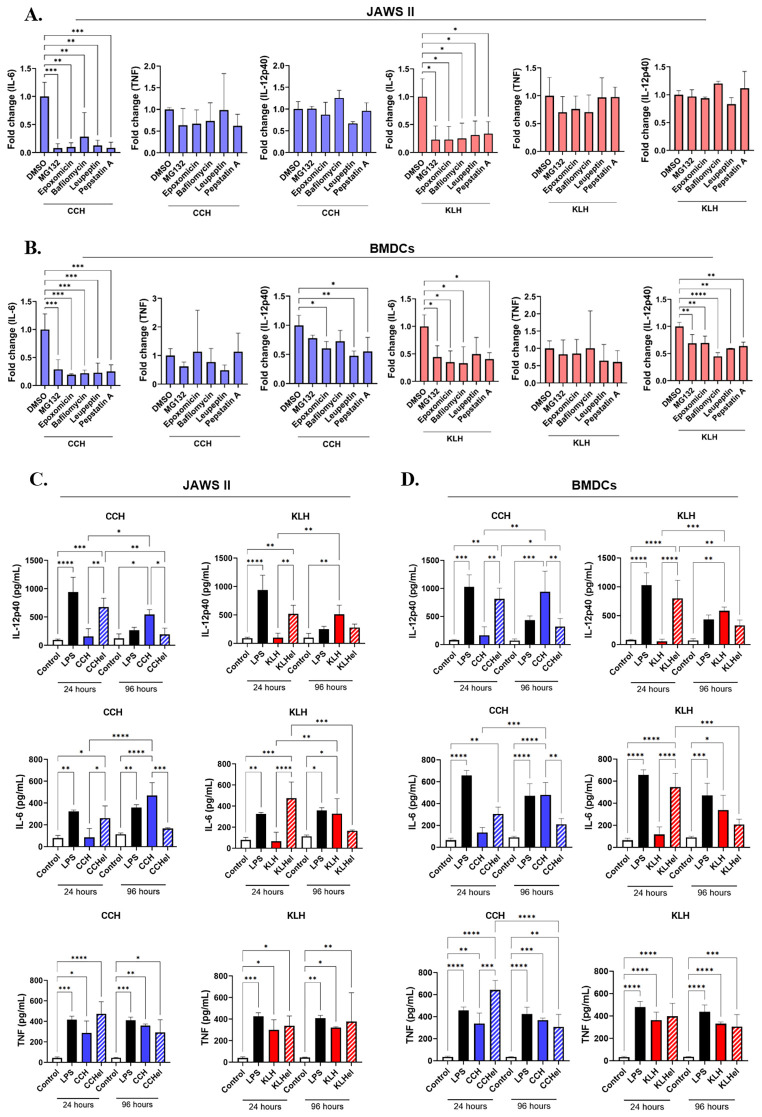
Pharmacological inhibition of lysosomes and proteasomes, as well as partial digestion of hemocyanins, impair cytokine secretion by APCs. JAWS II cells **(A)** or BMDCs **(B)** (2x10^5^) were pretreated with proteasomal inhibitors (MG132 and epoxomicin) or lysosomal inhibitors (bafilomycin, leupeptin, and pepstatin A) for 30 minutes and then stimulated with CCH (blue) or KLH (red) at 0.5 mg/mL. Cells pretreated with DMSO 0.001%, the vehicle of inhibitors, was used as the control. After 96 hours, supernatants were collected, and cytokines were quantified using a cytometric bead array (CBA) kit or BD OptEIA kits (ELISA). Similarly, JAWS II cells **(C)** or BMDCs **(D)** (2x10^5^) were stimulated with native or partially digested hemocyanins for 24 and 96 hours and supernatants were analyzed as mentioned by CBA or ELISA. For all figures, bar graphs show the mean ± SD of four independent experiments. For **(A, B)**, data shown as fold-change, where the cytokine secretion for each inhibitor was normalized against the values of the DMSO condition. For **(C, D)**, controls (LPS and culture medium) are the same because CCH and KLH data were obtained from the same experiment. Statistical analyses were performed by Kruskal-Wallis, where *<0.05, **p<0.01, ***p<0.001, ****p<0.0001.

Subsequent cytokine analyses, using the IC_50_ concentration of lysosomal and proteasomal inhibitors, revealed a significant reduction in the secretion of IL-6 by JAWS II cells (**Figure 7A**). These effects were similar for stimulation with both CCH and KLH. In the case of BMDCs, lysosome and proteasome inhibitors showed comparable effects on IL-6 and IL-12p40 (**Figure 7B**). Notably, the hemocyanin-dependent TNF secretion remained unaffected at all conditions tested, indicating a stimulatory profile that is independent of hemocyanin processing. Similarly, partially digested hemocyanins with elastase (CCHel and KLHel) induced the secretion of IL-6 and IL-12p40 within 24 hours, displaying an earlier response compared to native proteins, both in JAWS II and BMDCs (**Figures 7C, D**). The molecular weight of these preparations was below 50 kDa as confirmed by SDS-PAGE (**Supplementary Figures 7A, B**). These results indicate that the intracellular degradation of hemocyanins and oligomeric complexity are linked to the delayed proinflammatory effects in our APC models. Overall, the use of pharmacological inhibitors significantly reduced cytokine production in CCH- and KLH-treated APCs, highlighting the importance of intracellular processing in the induction of the late proinflammatory effects.

## 4 Discussion

The antigen processing of small proteins, such as OVA, has been extensively studied. However, similar research on larger and structurally stable oligomeric metalloglycoproteins, such as hemocyanins, remains limited (2, 65). Understanding how the protein complexity affects their immunogenicity is crucial for designing improved hemocyanin-based immunotherapies, as this property influences the processing and presentation of antigenic peptides by MHC molecules (45, 66).

Previous studies suggested a delayed degradation of CCH by BMDCs; however, as a monoclonal antibody was used in a similar analysis, only one low-molecular band was detected (41). Here, we used an anti-CCH serum, and several band patterns were deployed. Additionally, the proinflammatory effects of CCH and KLH have been analyzed *in vitro* on peritoneal macrophages after 24 and 48 hours, showing that CCH has a lower effect compared with KLH (67). With this information and utilizing two models of DCs, we aimed to delve deeper into the pro-inflammatory effects induced by hemocyanins by extending the analysis time and complementing it with an examination of their intracellular destination. The results show that hemocyanins stimulate an early TNF response, followed by delayed cytokine peaks of IL-12p40, IL-6, and TNF and upregulation of CD80 and CD86. Due to differences in assay timing, these hemocyanin-dependent effects on APCs may have been undetected in previous studies. Hence, our findings support the already demonstrated persistent Th1 response induced by hemocyanins, which may provide beneficial effects in antibacterial and antifungal immunity as well as in their antitumor, carrier, and adjuvant applications (68). Moreover, our findings demonstrate that hemocyanins maintain APCs in an actively cytokine producing state without the earlier cytotoxic effects of LPS, with TNF, IL-6, and IL-12p40 being the main contributors to the differences between CCH and KLH profiles. Consequently, the immune response triggered by the hemocyanins establishes a balanced inflammation, resulting in a beneficial bystander activator effect on mammals’ immune systems.

We used BMDCs and the JAWS II cell line, which had not been previously tested with hemocyanins. BMDCs are more representative of the *in vivo* behavior of DCs, while JAWS II provides reproducibility and enables the study of specific effects in a more controlled cellular system. Combining the two models helps us understand how hemocyanins are processed in different cellular contexts. Here, we detected basally high levels of MHC-I, an inducible increase in MHC-II and CD86, and the secretion of IL-12 by JAWS II, consistent with their reported phenotype (53). However, we obtained a smaller percentage of CD11c^+^ cells, which might be associated with the high concentration of serum required for their culture or with the method of cell harvesting, considering that JAWS II cells are grown in two fractions, adherent and non-adherent (69). Hence, CD11c levels may vary according to culture conditions, and we interpret the results from JAWS II as indicative of APCs’ effects, encompassing both iDC and undifferentiated monocyte populations. For BMDCs, the expression markers were consistent with those previously observed by our group (38, 70).

Hemocyanins exhibited comparable effects in JAWS II and BMDCs. Indeed, Jiang et al., showed that JAWS II could effectively replace BMDCs in a *Chlamydia* infection model (53). Our studies with CCH and KLH revealed that JAWS II cells do not express the endocytic receptor MR and exhibit a modest expression of TLR4, whose activation promotes TNF, IL-6, and IL-12. Both MR and TLR4 play a relevant role in hemocyanin immunity. In turn, JAWS II cells express SIGNR1, which is reported to promote the production of IL-6 and TNF, and SIGNR5, which promotes the production of IL-6, TNF, and IL-12. Hence, JAWS II would be a model for studying the effects of hemocyanins on DC-SIGN homologs in the context of hemocyanin recognition and endocytosis (37). It is important to note that pharmacological inhibition experiments yield a partial reduction in cytokine secretion, which several reasons can explain. First, inhibitors may exhibit transient effects that are not fully effective after 96 hours. Second, as hemocyanins have multiligand properties, alternative pathways that are not influenced by inhibitors may allow cytokine production to continue. Finally, working concentrations were set to the IC_50_ of IL-6 secretion, and inhibition of TNF and IL-12p40 may require higher concentrations; however, working values ensure a partial reduction without nonspecific or cytotoxic effects.

Additionally, both types of APCs exhibited a similar delayed hemocyanin-dependent proinflammatory peak, with upregulation of CD80 and CD86, suggesting that the late effects are reproducible across our APC models. The slow degradation of hemocyanin was comparable in both cases; however, significant differences were observed in colocalization between CCH and KLH with integrin α4. Finally, proteasomal and lysosomal inhibitors had comparable effects on the impairment of IL-6 secretion and, strikingly, they did not decrease TNF secretion; the possible effects of hemocyanins on the regulation of TNF mRNA had previously been discussed in murine macrophages, considering the transcriptional regulation, i.e., mRNA stability and protein shedding (67, 71, 72). We acknowledge that the use of non-parametric analysis may reduce the significance of the differences found. Still, it was used to avoid normality assumptions and provide a more conservative assessment of the data. Thus, cell lines like JAWS II serve as valuable tools for hemocyanin immunity.

After characterizing the kinetics of hemocyanin-induced cytokine secretion and costimulatory molecule upregulation, we hypothesized that their complex conformational stability could influence their long-term effects. Hemocyanins colocalize with C-type lectin receptors within the first 10 minutes of incubation and are internalized within less than 1 hour (33, 37, 41). Therefore, the prolonged effects are not due to delayed recognition or internalization by APCs but rather involve intracellular trafficking of hemocyanins. In a model of BMDCs stimulated with OVA alone or with OVA plus LPS, it was observed that lysosomal processing was delayed following TLR4 activation (73); however, as OVA is rapidly processed, it still localized to lysosomes within 24 hours of incubation. Typically, OVA colocalization with LAMP1 and cytokine secretion are analyzed after 0.5 and 4 hours post-stimulation (74). In contrast, OVA-targeted IgG complexes, unlike OVA alone, persisted in BMDCs for up to 4 days, with around 50% remaining after 96 hours. This suggests that oligomeric proteins, which are internalized in a receptor-dependent manner, are slowly processed as these complexes are stored in intracellular antigen depots that do not colocalize with MHC-II nor with TAP (75). Consequently, the slow processing of hemocyanins and their delayed colocalization with LAMP1 indicate that they may have been previously localized in intracellular depots. Further research may explore the kinetics of earlier events, such as colocalization with early endosomes. It is well-established that antigens with decreased susceptibility to lysosomal degradation elicit stronger immune responses (48). It is important to note that, in the case of vaccines, this is especially important because using an antigen with delayed kinetics can enhance the magnitude, quality, and persistence of antibody responses (76).

LAMP1 is commonly recognized and used as a lysosomal marker; however, it has also been found in various other intracellular compartments, including late endosomes, the trans-Golgi network, perinuclear regions, and even on the cell membrane during sorting events (77). Moreover, LAMP1 has been detected in the same intracellular regions as Rab5 and Rab7. However, this occurs in a cell-specific manner, suggesting that endosome maturation is not a determining factor for lysosome fusion (78). In our observations, LAMP1 was widely distributed among the APCs, consistent with its potential heterogeneous distribution, until 96 hours post-stimulation, when we noticed LAMP1 localized in areas where hemocyanins were located. Therefore, we propose that the initial lack of colocalization between hemocyanins and LAMP1 not only indicates an absence of lysosomal localization and rules out a destination to late endosomal fusion vesicles. This supports the idea that storage depots play a role in contributing to a slow degradation process.

Notably, our research reveals the role of proteasomes in the intracellular processing of hemocyanins for the first time. This was demonstrated through colocalization studies and pharmacological inhibitors, including MG132 and epoxomicin. It is widely accepted that when stimulated with agents such as LPS or proinflammatory cytokines, mature DCs upregulate the immunoproteasome subunits, which are more efficient in protein degradation. In contrast, the half-life of constitutive proteasomes exceeds 96 hours (79). Hence, significant changes in proteasome proportions may only be observable after several days, during which the spectrum of class-I peptides may change. Supporting this idea, a mouse model lacking immunoproteasome subunits showed significant differences in the repertoire of MHC-I presented peptides, but no significant changes were observed in those restricted to MHC-II (80). However, it has also been suggested that immunoproteasomes are constitutively expressed in DCs and that they cannot process antigens generated by the constitutive proteasome (81). In our study, we detected LAMP2 and α4 in JAWS II and BMDCs, indicating their basal expression in resting APCs. Hemocyanin was found to promote their intracellular reorganization. Based on the colocalization coefficients, we expect that JAWS II would generate only immunoproteasome peptides, while BMDCs would accumulate both constitutive and immunoproteasome peptides. Moreover, in BMDCs, proteasomes and immunoproteasomes can be translocated to phagosomes and endosomes, producing TAP-independent peptides for cross-presentation (82). In fact, in cross-presenting DCs, phagosomes/endosomes maintain a neutral pH in their lumen to preserve antigens, which also provides optimal conditions for proteasomal function (82–84). Thus, since hemocyanins undergo receptor-mediated endocytosis and macropinocytosis, which facilitate multivesicular intracellular trafficking, and since, in our study, we observed hemocyanins and proteasomes were colocalized in compartmentalized spots, proteasomes would be translocated inside phagosomes/endosomes, thereby contributing to the hemocyanin processing. This would also explain the kinetics of our findings in BMDCs, where immunoproteasomes displayed earlier colocalization with hemocyanins compared to lysosomes. Furthermore, proteins can escape from endosomes into the cytosol through transporters or because of a ROS-dependent leaky membrane, and these mechanisms should not be discarded (85). Furthermore, the role of immunoproteasomes in protein degradation also influences transcriptomic modulation, including STAT, IRF, and NF-kB pathways (86). Thus, proteasomal degradation of hemocyanins contributes to generating the immunopeptidome, while the potential effects on cell transcription should also be considered.

Our findings highlight the extended immune responses induced by CCH and KLH. PBAs, such as hemocyanins, activate multiple mechanisms in APCs, including the stimulation of immune receptors and the induction of a humoral response against haptens and co-administered molecules (9, 33, 37, 38, 68). The contribution of lysosomal degradation to the adjuvant effects of hemocyanins, had been suggested by OT-I and OT-II murine models, where bafilomycin decreased T-cell priming (5). Hence, further studies should focus on characterizing the intracellular processing and of the co-administered antigens, particularly examining the dual role of hemocyanins as carriers and adjuvants. For example, the immune effects of Flagellin, a well-known bacterial PBA that targets TLR5, have been evaluated as both a carrier and an adjuvant simultaneously, using the native Flagellin or specific sequences of the protein. The results showed robust immunity against antigens from vaccinia virus, *Plasmodium falciparum*, HIV, and even cocaine, which includes the activation of humoral response and complement-dependent immunity (87–90). Thus, our results suggest the investigation into the dual role of hemocyanins as carriers and adjuvants, using a wide range of antigens, because hemocyanins are destined for both lysosomes and proteasomes, which may facilitate the targeting of antigens to these compartments, resulting in a prolonged and sustained accumulation of antigenic peptides. The multiligand characteristics of hemocyanins may enhance their trafficking to multiple endocytic compartments, resulting in a diverse immunopeptidome (33, 37, 38). The slow processing antigen dependent on hemocyanins could enhance the presentation of these antigens by MHC molecules over an extended period. This process is regulated by the quantity and quality of the peptides, i.e., the concentration and affinity of peptide-MHC interaction (91).

Remarkably, hemocyanins have an impressive ability to address a common limitation of typical adjuvants: a poor activation of CD8^+^ cytotoxic T lymphocytes (CTLs) (68, 85). Previous studies have shown that hemocyanins enhance CD8^+^ T-cell priming when used as PBAs (5). In this study, we demonstrate that they are localized with immunoproteasomes, which are related and typically associated with MHC-I antigen presentation. Consequently, hemocyanins hold significant potential for facilitating the cross-presentations of antigens in Th1 responses, particularly in infectious diseases or cancer immunotherapy (85, 92). Furthermore, future research will explore the structural insights of hemocyanins and the potential use of specific subunits/polypeptides to mitigate the limitations associated with natural products (2, 28).

In summary, we demonstrated that two types of murine APCs slowly process two well-documented hemocyanins. This processing involves key roles from lysosomal and proteasomal degradation, which coincide with a delayed proinflammatory cytokine and costimulatory molecule response. Our study enhances the understanding of how hemocyanin affects APC responses to complex, oligomeric glycoproteins. We identified additional cytokines secreted in a hemocyanin-dependent manner, conducted long-term protocols that had not been addressed before, and examined the contribution of well-characterized proteolytic machineries. We also validated these effects by comparing them with OVA. Since hemocyanins are safe, widely used glycoproteins that induce a robust Th1 and a potential Th17 bias, they serve as an effective tool for enhancing immunity against bacterial, viral, and fungal infections, as well as in strategies against certain types of cancer. Their slow processing, prolonged antigen presentation, and potential for cross-presentation further support their use as novel and effective PBAs in immunotherapeutic applications.

## 5 Conclusions

The results show a correlation between the late proinflammatory response and proteolytic processing times of two distinct hemocyanins compared to OVA.

CCH and KLH initiate an early TNF response, followed by a substantial increase in IL-12p40, IL-6, and TNF at 96 hours, a response significantly greater than that observed with OVA.

The slow processing and delayed colocalization of hemocyanins with LAMP1 suggest that these proteins are likely stored in intracellular depots and gradually degraded over time, contributing to their sustained immune-stimulatory effects.

This study is the first to demonstrate the crucial role of proteasomes in the intracellular processing of hemocyanins, highlighting their importance in antigen degradation. The reorganization of immunoproteasomes in APCs following hemocyanin stimulation suggests an enhanced and sustained presentation of MHC-I peptides, which could prolong the duration of immune activation.

Hemocyanins have unique effects that make them potential novel adjuvants in vaccine formulations. Their ability to sustain immune activation could provide an advantage in inducing long-lasting protection against infections, promoting a robust and durable Th1-mediated immune response.

Finally, this research provides valuable insights into how complex oligomeric glycoproteins extend and enhance their proinflammatory effects on APCs.

## Data Availability

The raw data supporting the conclusions of this article will be made available by the authors, without undue reservation.

## Supplementary material

Supplementary Figure 1 Hemocyanin-dependent proinflammatory response in APCs. JAWS II cells **(A)** or BMDCs **(B)** (2x10^5^) were stimulated with CCH (blue) and KLH (red) at a final concentration of 0.5 mg/mL. LPS (black, 1 ng/mL) was used as the positive control. Ovalbumin (OVA, grey, 0.5 mg/mL) was used as a model antigen. Unstimulated cells were used as the negative control (Control, white). After 24, 72, and 96 hours, supernatants were collected, and cytokines were quantified using a cytometric bead array (CBA) kit or BD OptEIA kits (ELISA). Bar graphs show the mean ± SD of three independent experiments. Statistical analyses were performed by Kruskal-Wallis, where the samples were compared against the negative control (Control, white) and *p<0.05.

Supplementary Figure 2 CCH and KLH do not elicit cytotoxic effects up to 96 hours post-incubation in APCs. JAWS II **(A)** or BMDCs **(B)** (1x10^5^) were stimulated with CCH (blue) and KLH (red) at a final concentration of 0.5 mg/mL. LPS (black, 1 ng/mL) was used as the positive control. Unstimulated cells were used as the negative control (Control, white). After 24–120 hours, cell viability was assessed using AlamarBlue staining. Bar graphs show the mean ± SD of at least three independent experiments. Statistical analyses were performed by Kruskal-Wallis where the samples were compared against the negative control (Control, white). *<0.05. **p<0.01.

Supplementary Figure 3 Characterization of the JAWS II cell line and BMDCs. JAWS II **(A)** or BMDCs **(B)** (1x10^5^) were stained with the following antibodies: anti-CD11C-PE, anti-MR-FITC, anti-TLR4-FITC, anti-SIGNR1-Alexa647, and anti-SIGNR5-Alexa488. Additionally, cells were incubated with CCH- and KLH-Alexa650 for 1 hour. Upper panels show representative histograms, and bar graphs show the mean ± SD of three independent experiments. Analyses were performed using Mann-Whitney, where *p<0.05.

Supplementary Figure 4 CCH and KLH had differential effects on MHC-I and MHC-II in APCs. JAWS II **(A)** or BMDCs **(B)** (2x10^5^) were stimulated with CCH (blue), KLH (red) or OVA (grey) at a final concentration of 0.5 mg/mL. Lipopolysaccharide from *E. Coli* (LPS, black, 1 ng/mL) was used as the positive control. Unstimulated cells were used as the negative control (Control, white). After 24, 72 and 96 hours, cells were stained with anti-MHC-I-PE or anti-MHC-II-Alexa647 antibodies. Bar graphs show the mean ± SD of three independent experiments. Statistical analyses were performed by Kruskal-Wallis, where the samples were compared against the negative control and *p<0.05. ***p<0.001.

Supplementary Figure 5 Standardization of inhibitors. JAWS II **(A)** or BMDCs **(B)** (2x10^5^) were pretreated with increasing concentrations of inhibitors (0–40 nM) and then with CCH or KLH (0.5 mg/mL). Ninety-six hours later, cell viability was assessed by AlamarBlue (upper panels), and the fold change of IL-6 or IL-12p40 secretion was quantified (lower panels). Graphs show the mean ± SD of three independent experiments. IC_50_ values were estimated according to cytokine secretion, considering the ranges where cells remained viable.

Supplementary Figure 6 Pharmacological inhibitors do not induce nonspecific effects in APCs. JAWS II (left panels) or BMDCs (right panels) (2x10^5^) were pretreated with pharmacological inhibitors for 30 minutes and then incubated with culture medium only **(A)** or with LPS (1 ng/mL) **(B)**. Ninety-six hours later, the secretion of IL-6 or IL-12p40 was quantified using BD OptEIA kits. Graphs show the mean ± SD of three independent experiments. Data shown as fold-change after normalizing the cytokine secretion promoted by each inhibitor against the DMSO values. Statistical analyses were performed using the Kruskal-Wallis, where the samples were compared against the negative control and **p<0.01.

Supplementary Figure 7 Partial proteolysis of hemocyanins. SDS-PAGE analysis of CCH **(A)** and KLH **(B)** before and after elastase digestion. Lane 1: molecular weight marker (MW). Lane 2: native hemocyanin. Lane 3: elastase-digested hemocyanin (CCHel and KLHel). Representative images of three independent experiments.
