# Supplementary figures and images for: Conformational stability of hemocyanins regulates their lysosomal and proteasomal degradation, influencing their pro-inflammatory effects on mammalian antigen-presenting cells
Source: Front Immunol. 2025 Dec 1;16:1603070. doi: 10.3389/fimmu.2025.1603070 (PMC12715429; doi:10.3389/fimmu.2025.1603070)

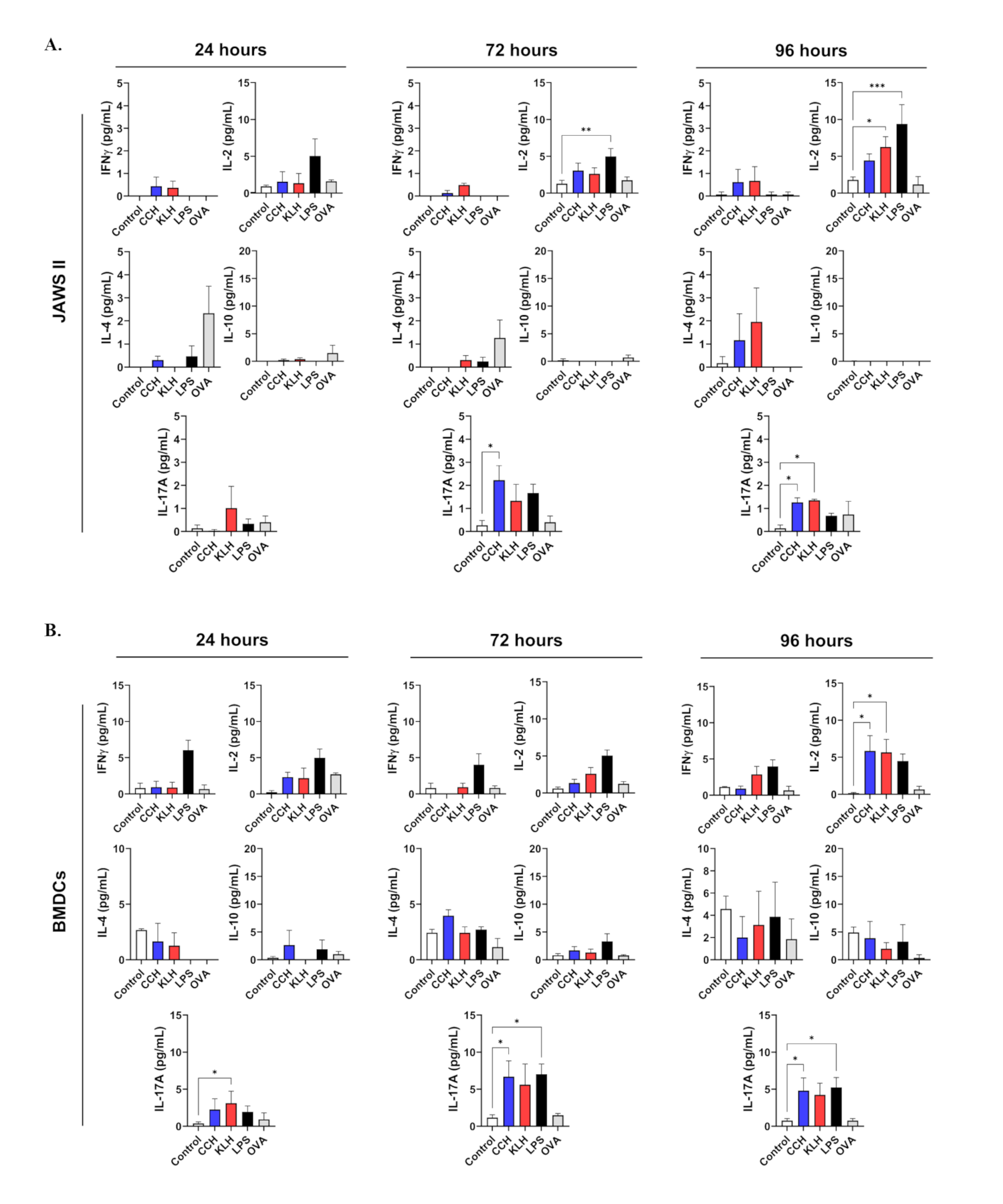

Supplement: Supplementary Figure 1 — Hemocyanin-dependent proinflammatory response in APCs. JAWS II cells (A) or BMDCs (B) (2x10^5^) were stimulated with CCH (blue) and KLH (red) at a final concentration of 0.5 mg/mL. LPS (black, 1 ng/mL) was used as the positive control. Ovalbumin (OVA, grey, 0.5 mg/mL) was used as a model antigen. Unstimulated cells were used as the negative control (Control, white). After 24, 72, and 96 hours, supernatants were collected, and cytokines were quantified using a cytometric bead array (CBA) kit or BD OptEIA kits (ELISA). Bar graphs show the mean ± SD of three independent experiments. Statistical analyses were performed by Kruskal-Wallis, where the samples were compared against the negative control (Control, white) and *p<0.05. [file Image1.tif]

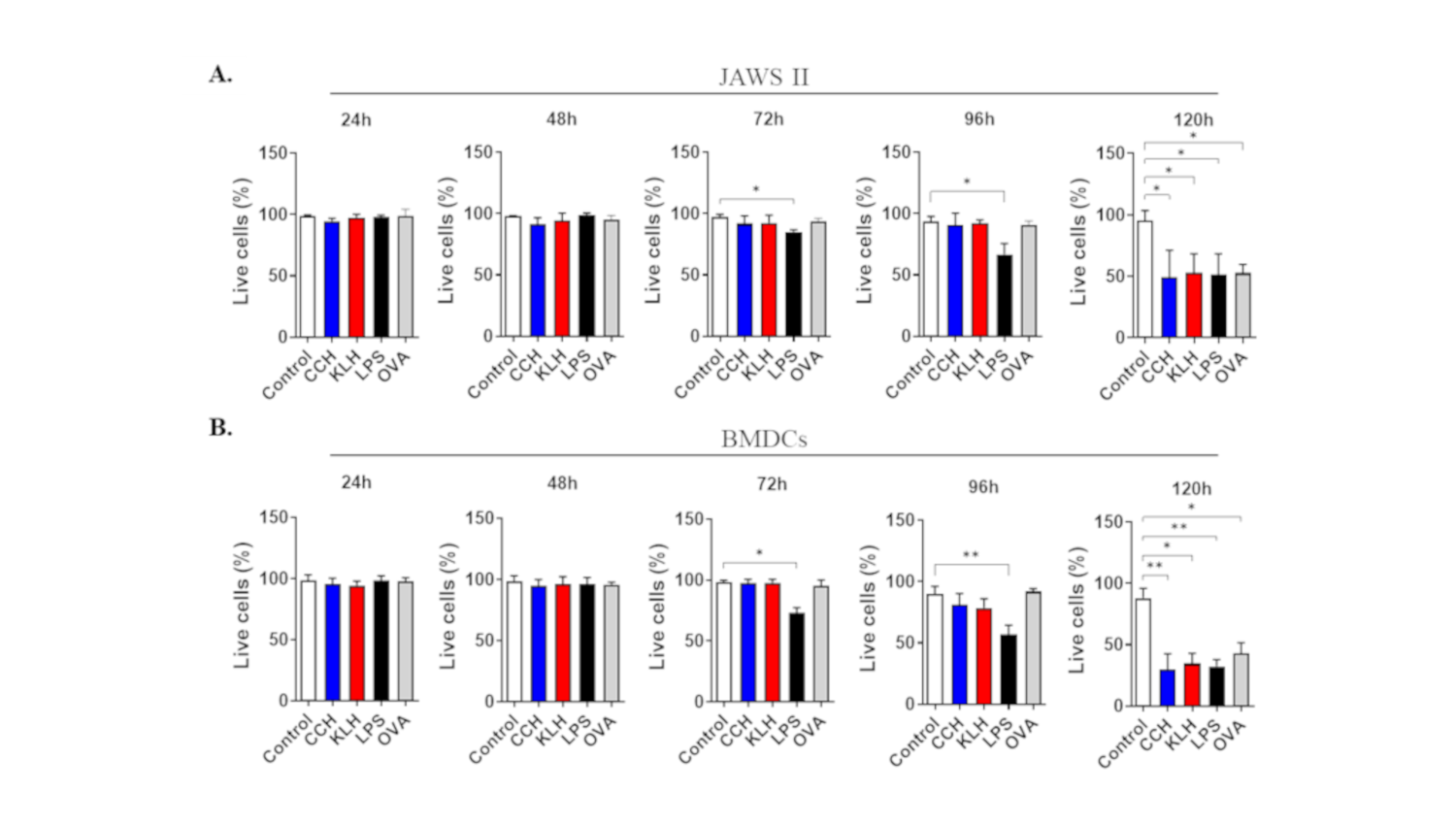

Supplement: Supplementary Figure 2 — CCH and KLH do not elicit cytotoxic effects up to 96 hours post-incubation in APCs. JAWS II (A) or BMDCs (B) (1x10^5^) were stimulated with CCH (blue) and KLH (red) at a final concentration of 0.5 mg/mL. LPS (black, 1 ng/mL) was used as the positive control. Unstimulated cells were used as the negative control (Control, white). After 24–120 hours, cell viability was assessed using AlamarBlue staining. Bar graphs show the mean ± SD of at least three independent experiments. Statistical analyses were performed by Kruskal-Wallis where the samples were compared against the negative control (Control, white). *<0.05. **p<0.01. [file Image2.tif]

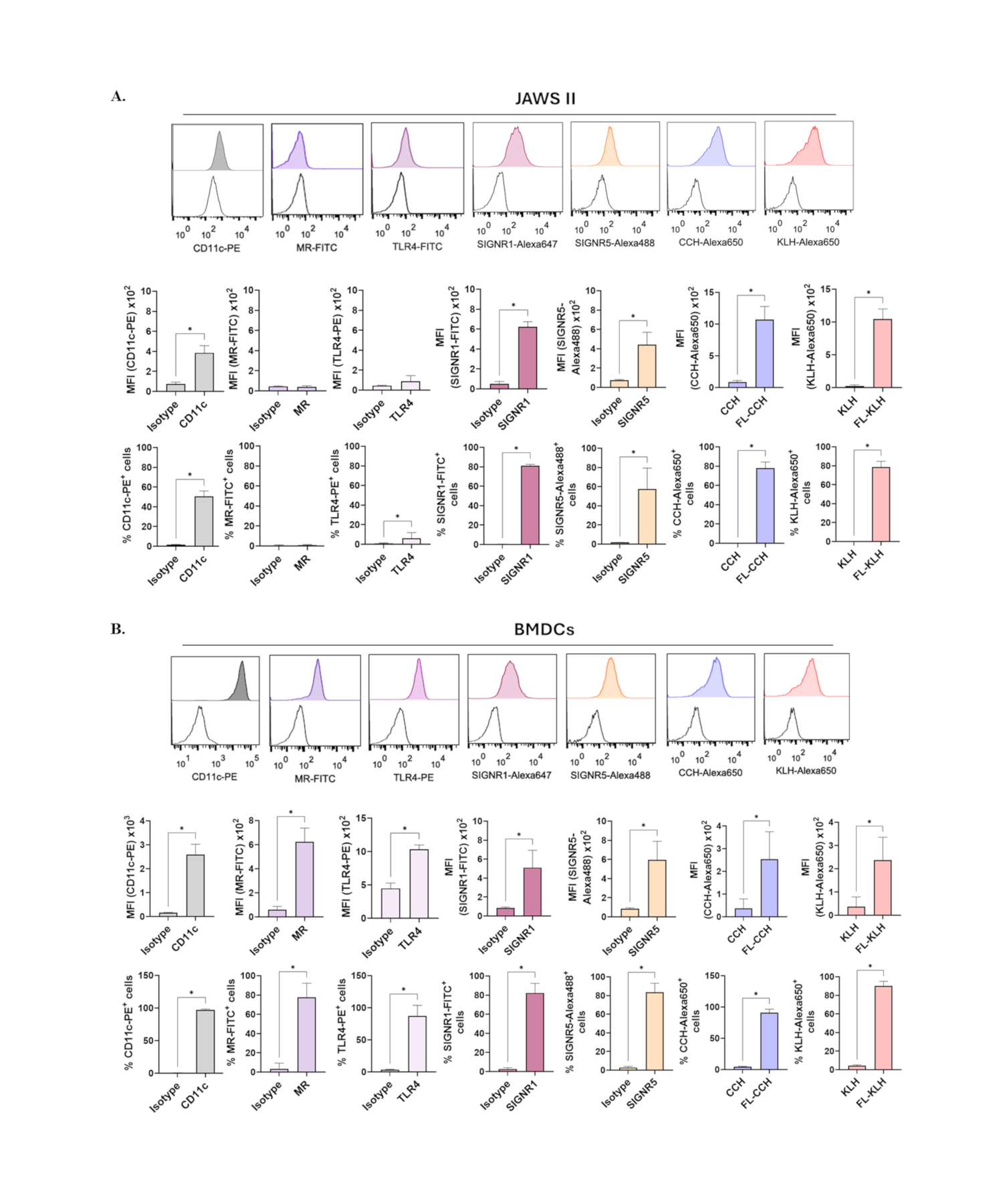

Supplement: Supplementary Figure 3 — Characterization of the JAWS II cell line and BMDCs. JAWS II (A) or BMDCs (B) (1x10^5^) were stained with the following antibodies: anti-CD11C-PE, anti-MR-FITC, anti-TLR4-FITC, anti-SIGNR1-Alexa647, and anti-SIGNR5-Alexa488. Additionally, cells were incubated with CCH- and KLH-Alexa650 for 1 hour. Upper panels show representative histograms, and bar graphs show the mean ± SD of three independent experiments. Analyses were performed using Mann-Whitney, where *p<0.05. [file Image3.tif]

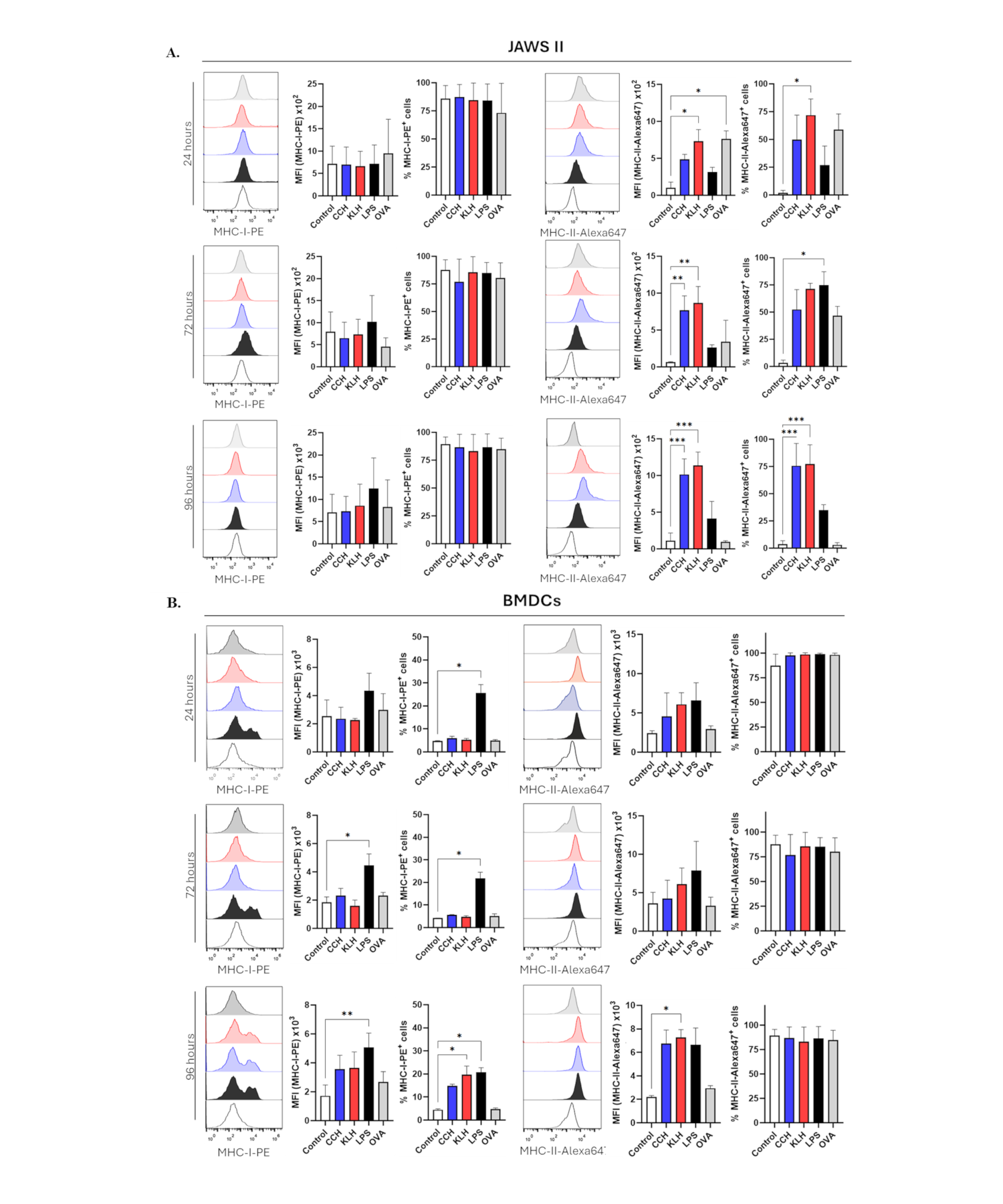

Supplement: Supplementary Figure 4 — CCH and KLH had differential effects on MHC-I and MHC-II in APCs. JAWS II (A) or BMDCs (B) (2x10^5^) were stimulated with CCH (blue), KLH (red) or OVA (grey) at a final concentration of 0.5 mg/mL. Lipopolysaccharide from E. Coli (LPS, black, 1 ng/mL) was used as the positive control. Unstimulated cells were used as the negative control (Control, white). After 24, 72 and 96 hours, cells were stained with anti-MHC-I-PE or anti-MHC-II-Alexa647 antibodies. Bar graphs show the mean ± SD of three independent experiments. Statistical analyses were performed by Kruskal-Wallis, where the samples were compared against the negative control and *p<0.05. ***p<0.001. [file Image4.tif]

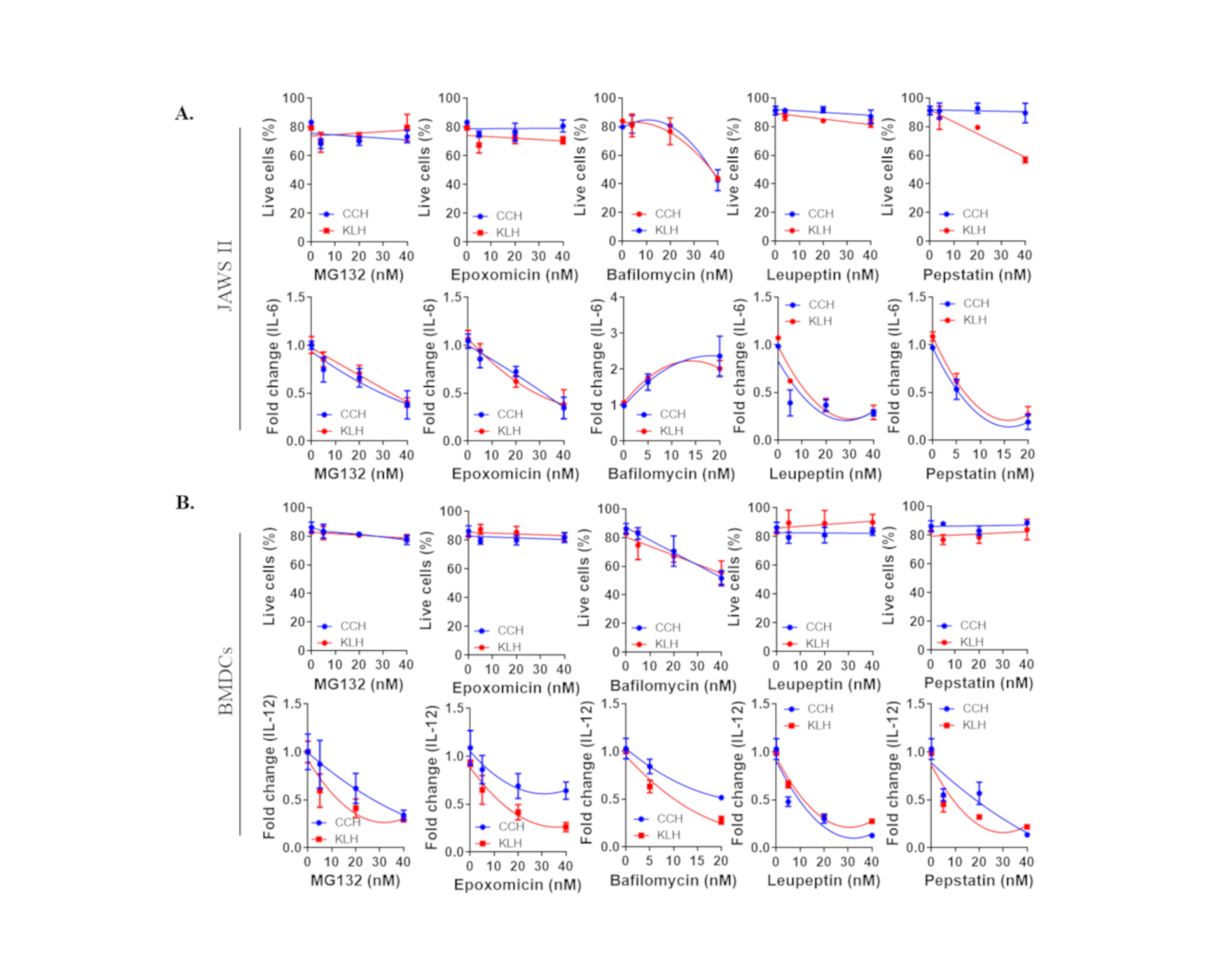

Supplement: Supplementary Figure 5 — Standardization of inhibitors. JAWS II (A) or BMDCs (B) (2x10^5^) were pretreated with increasing concentrations of inhibitors (0–40 nM) and then with CCH or KLH (0.5 mg/mL). Ninety-six hours later, cell viability was assessed by AlamarBlue (upper panels), and the fold change of IL-6 or IL-12p40 secretion was quantified (lower panels). Graphs show the mean ± SD of three independent experiments. IC50 values were estimated according to cytokine secretion, considering the ranges where cells remained viable. [file Image5.tif]

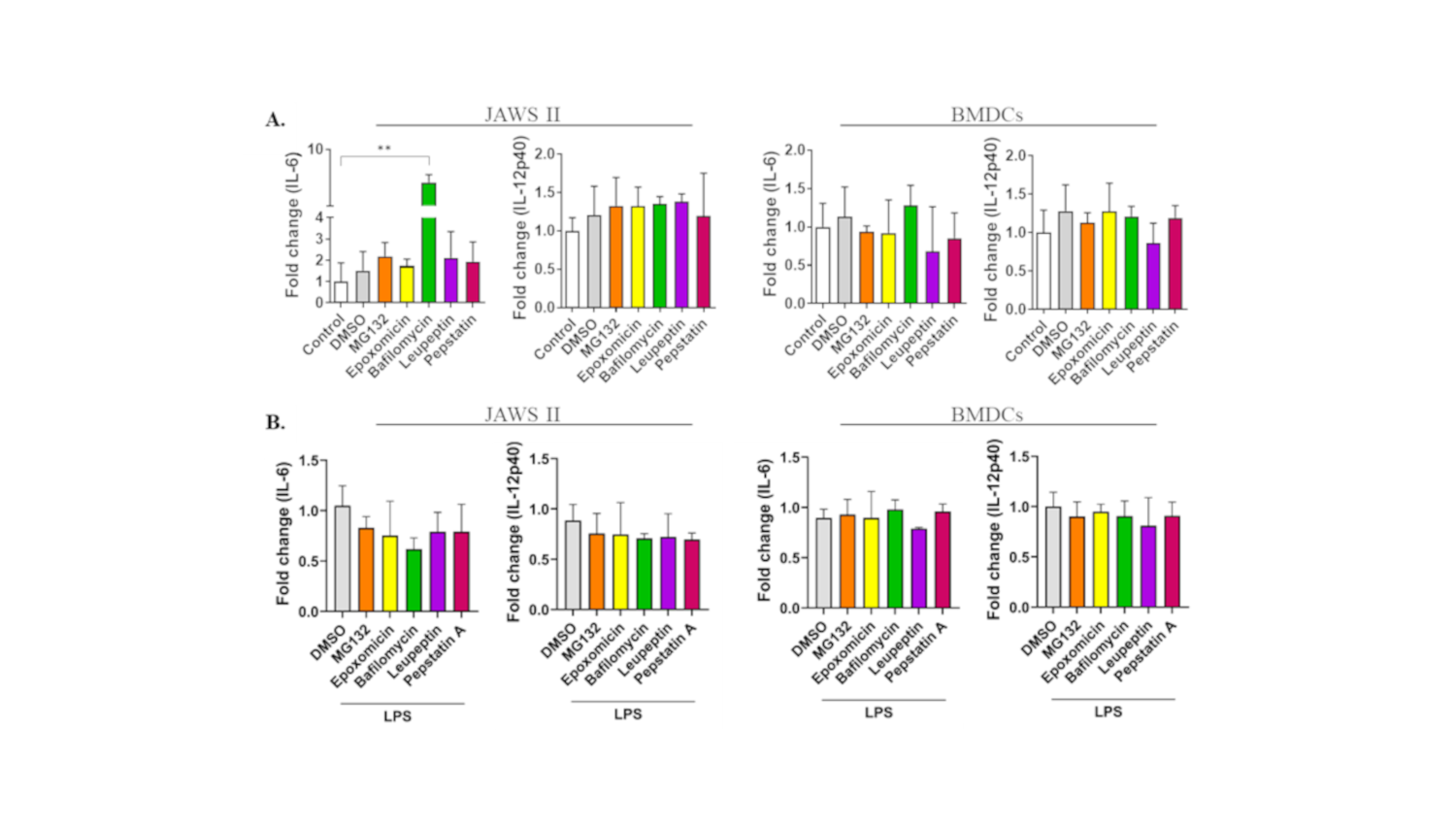

Supplement: Supplementary Figure 6 — Pharmacological inhibitors do not induce nonspecific effects in APCs. JAWS II (left panels) or BMDCs (right panels) (2x10^5^) were pretreated with pharmacological inhibitors for 30 minutes and then incubated with culture medium only (A) or with LPS (1 ng/mL) (B). Ninety-six hours later, the secretion of IL-6 or IL-12p40 was quantified using BD OptEIA kits. Graphs show the mean ± SD of three independent experiments. Data shown as fold-change after normalizing the cytokine secretion promoted by each inhibitor against the DMSO values. Statistical analyses were performed using the Kruskal-Wallis, where the samples were compared against the negative control and **p<0.01. [file Image6.tif]

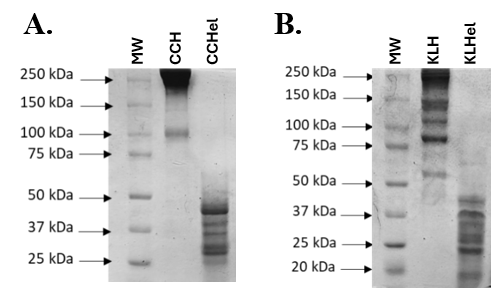

Supplement: Supplementary Figure 7 — Partial proteolysis of hemocyanins. SDS-PAGE analysis of CCH (A) and KLH (B) before and after elastase digestion. Lane 1: molecular weight marker (MW). Lane 2: native hemocyanin. Lane 3: elastase-digested hemocyanin (CCHel and KLHel). Representative images of three independent experiments. [file Image7.tif]
