# Supplementary material for: Conformational stability of hemocyanins regulates their lysosomal and proteasomal degradation, influencing their pro-inflammatory effects on mammalian antigen-presenting cells
Source: Front Immunol. 2025 Dec 1;16:1603070. doi: 10.3389/fimmu.2025.1603070 (PMC12715429; doi:10.3389/fimmu.2025.1603070)
Supplement: Supplementary file 8 [file Supplementaryfile1.docx]

**Supplementary Table 1. Loadings from Principal Component Analysis (PCA) and Clustering of cytokine response data.**

|  | **PCA loadings JAWS II** | | **PCA loadings BMDCs** | |
| --- | --- | --- | --- | --- |
| **Cytokine** | **PC1 (45.2%)** | **PC2 (16.9%)** | **PC1 (56.7%)** | **PC2 (16.2%)** |
| IFN | 0.1785347 | 0.0946676 | 0.3549564 | 0.1638529 |
| IL-2 | 0.3943932 | 0.0888442 | **0.4520480** | 0.0846670 |
| IL-4 | 0.1134170 | **-0.8445643** | 0.0055761 | **-0.8561043** |
| **IL-6** | **0.4465254** | **-0.3744780** | **0.4701577** | 0.0452502 |
| **IL-12p40** | **0.4970393** | 0.0002207 | **0.4389920** | 0.0790067 |
| IL-17A | 0.3604439 | 0.2633278 | 0.3159661 | **-0.4740773** |
| **TNF** | **0.4726132** | 0.2455207 | **0.3950072** | 0.0055033 |
